# Strategies to reinvigorate exhausted CD8^+^ T cells in tumor microenvironment

**DOI:** 10.3389/fimmu.2023.1204363

**Published:** 2023-06-16

**Authors:** Qianting Guan, Meiwen Han, Qinghao Guo, Fangfei Yan, Ming Wang, Qin Ning, Dong Xi

**Affiliations:** Department and Institute of Infectious Disease, Tongji Hospital, Tongji Medical College and State Key Laboratory for Diagnosis and Treatment of Severe Zoonotic Infectious Disease, Huazhong University of Science and Technology, Wuhan, Hubei, China

**Keywords:** exhausted CD8 + T cells, tumor microenvironment, immune checkpoint blockade, transcription factor-based therapy, epigenetic therapy, metabolism-based therapy, cytokine therapy

## Abstract

CD8^+^ T cell exhaustion is a stable dysfunctional state driven by chronic antigen stimulation in the tumor microenvironment (TME). Differentiation of exhausted CD8^+^ T cells (CD8^+^ TEXs) is accompanied by extensive transcriptional, epigenetic and metabolic reprogramming. CD8^+^ TEXs are mainly characterized by impaired proliferative and cytotoxic capacity as well as the increased expression of multiple co-inhibitory receptors. Preclinical tumor studies and clinical cohorts have demonstrated that T cell exhaustion is firmly associated with poor clinical outcomes in a variety of cancers. More importantly, CD8^+^ TEXs are regarded as the main responder to immune checkpoint blockade (ICB). However, to date, a large number of cancer patients have failed to achieve durable responses after ICB. Therefore, improving CD8^+^ TEXs may be a breakthrough point to reverse the current dilemma of cancer immunotherapy and eliminate cancers. Strategies to reinvigorate CD8^+^ TEXs in TME mainly include ICB, transcription factor-based therapy, epigenetic therapy, metabolism-based therapy and cytokine therapy, which target on different aspects of exhaustion progression. Each of them has its advantages and application scope. In this review, we mainly focus on the major advances of current strategies to reinvigorate CD8^+^ TEXs in TME. We summarize their efficacy and mechanisms, identify the promising monotherapy and combined therapy and propose suggestions to enhance the treatment efficacy to significantly boost anti-tumor immunity and achieve better clinical outcomes.

## 1 Introduction

T cell exhaustion is a “locked” dysfunctional state of CD8^+^ T cells, commonly seen in chronic infections and cancers (1). CD8^+^ T cells have been stimulated by massive immunosuppressive signals including chronic TCR signaling, hypoxia, nutrient deficiency and pro-inflammatory cytokines, which worked together to induce T cell exhaustion in tumor microenvironment (TME) (2–4). Exhaustion is an independent differentiation state of T cells (5–7). Exhausted CD8^+^ T cells (CD8^+^ TEXs) have undergone extensive transcriptional and epigenetic regulations and metabolic reprogramming (2, 3, 5). Characteristics of CD8^+^ TEXs include limited proliferative capacity, cytotoxicity and cytokine production and the increased expression of several inhibitory receptors such as programmed cell death 1 (PD1), T cell immunoglobulin domain and mucin domain-3 (TIM3) (1, 5). It’s of great importance to reinvigorate CD8^+^ TEXs in TME. Preclinical studies and clinical cohorts have shown that there was a large amount of CD8^+^ TEXs in tumors, which was closely related to poor clinical outcomes in a variety of cancers (lung cancer, melanoma, liver cancer, etc.) (5, 8–11). In addition, T cell exhaustion decreased the efficacy of some treatments, especially the chimeric antigen receptor (CAR)-T cell therapy (12, 13). TME induced dysfunctional CAR-T cells, which was the main barrier of CAR-T cell therapy in solid tumors (5, 14–16). Furthermore, TEXs are the main responder to immune checkpoint blockade (ICB). However, the efficacy of ICB has been severely limited by drug resistance and low response rates (16, 17). TEXs may be a breakthrough point to improve the efficacy of ICB. It’s obvious that inhibiting or improving CD8^+^ TEXs has great therapeutic potential.

Over the past decade, considerable research has been conducted to elucidate strategies to suppress differentiation of exhaustion. Strategies including ICB, epigenetic therapy, transcription factor-based therapy, metabolism-based therapy and cytokine therapy, have been reported to be effective in improving CD8^+^ TEXs. These strategies significantly inhibited the development of terminal exhaustion in TEXs and improved anti-tumor immunity. These strategies target on different aspects of exhaustion progression. ICB and cytokine therapy mainly regulate the inflammatory pathway in TEXs while epigenetic therapy and transcription factor-based therapy regulate the expression of exhaustion-related genes. In addition, metabolism-based therapy greatly improves metabolic defeats in TEXs. Combination therapy such as “ICB + metabolism-based therapy” and “ICB + cytokine therapy” showed superior efficacy and better tumor control than anti-PD1 monotherapy (5, 18–21). In this review, we focus on the main strategies to reinvigorate exhausted CD8^+^ T cells in TME. We summarize their effects and mechanisms and propose suggestions for precision treatment and combination therapy, to provide bases for optimizing current anti-tumor treatment in clinical practice and achieving superior tumor control.

## 2 Differentiation of exhausted CD8^+^ T cells in tumor microenvironment

A comprehensive understanding of the features and differentiation of CD8^+^ TEXs plays an important role in the discovery of therapeutic targets and the application of treatment strategies. CD8^+^ TEXs have experienced unique transcriptional and epigenetic regulation and metabolic reprogramming (1). However, there are still a lot of unknowns in the development of T cell exhaustion in TME.

### 2.1 Characteristics of exhausted CD8^+^ T cell subsets

**Figure 1:**
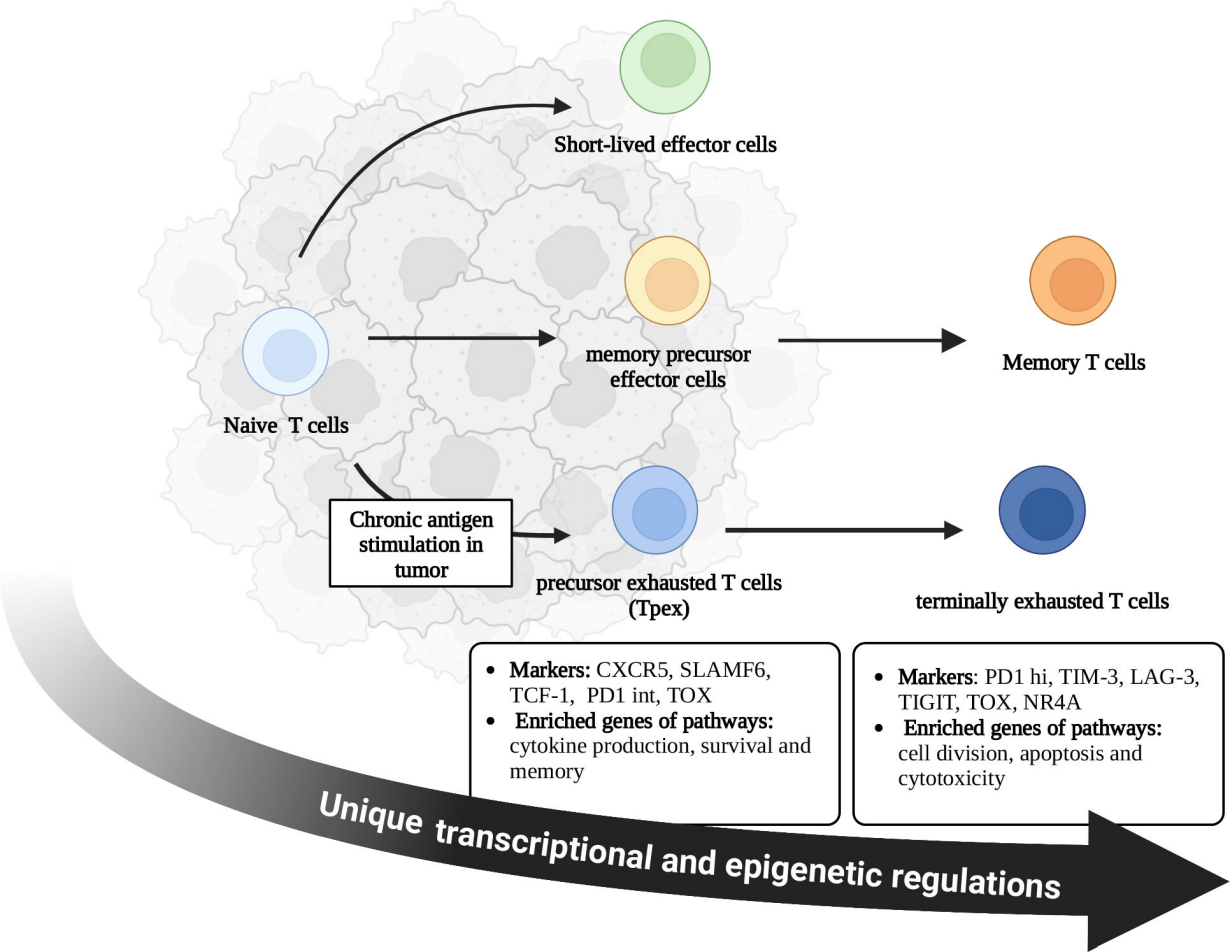
Differentiation of CD8^+^ T cells in tumor microenvironment. During acute infection, naive CD8^+^ T cells become activated and differentiate into short-lived effector cells or memory precursor effector cells. With the elimination of pathogens, most short-lived effector cells undergo apoptosis, while memory precursor effector cells survive and differentiate into memory CD8^+^ T cells. Memory T cells contribute to the long-term immunity against tumors. Persistent antigen exposure in tumor microenvironment alters the differentiation of CD8^+^ T cells toward exhaustion rather than memory, which is accompanied by unique transcriptional and epigenetic regulations. Exhausted T cells include Tpex and terminally exhausted T cells. Tpex were mainly distributed in tumor stromal region, lymph nodes, and peripheral blood, while terminally exhausted subsets were located in tumor. Chronic TCR signaling induces the differentiation of Naïve CD8^+^ T cells into Tpex in the TME, which further differentiate into terminally exhausted CD8^+^ T cells. Tpex in lymph nodes migrate to tumors in some situations such as after PD1/PDL1 blockade. There are different characteristics and functions between these two subsets. C-X-C chemokine receptor type 5 (CXCR5); Signaling lymphocyte activation molecule family member 6 (SLAMF6); T cell factor-1 (TCF-1); Thymocyte selection-associated high mobility group box protein (TOX); Nuclear receptor 4A (NR4A); Programmed cell death 1- intermediate (PD1-int); Programmed cell death 1- high (PD1-hi); T cell immunoglobulin domain and mucin domain-3 (TIM3); Lymphocyte activation gene-3 (LAG3); T cell immunoreceptor with immunoglobulin and ITIM domain (TIGIT).

Naïve CD8^+^ T cells differentiate into memory T cells, which exert long-term anti-tumor effects. However, persistent antigen exposure in TME has altered the differentiation of CD8^+^ T cells toward exhaustion (6, 22). There is considerable heterogeneity in CD8^+^ TEXs. CD8^+^ TEXs mainly included two distinct subsets: precursor exhausted T cells (Tpex) and terminally exhausted CD8^+^ T cells (1, 5). Tpex were mainly distributed in tumor stromal region, lymph nodes, and peripheral blood, while terminally exhausted subsets were located in tumor. Therefore, chronic TCR signaling induces the differentiation of Naïve CD8^+^ T cells into Tpex in the TME, which further differentiate into terminally exhausted CD8^+^ T cells. Tpex in lymph nodes migrate to tumors in some situations, such as after PD1/PDL1 blockade (**Figure 1**) (6, 22, 23). However, the decisive factor driving the exhaustion differentiation remained unknown.

Tpex generally expressed T cell factor-1 (TCF-1), PD1 (intermediate level), C-X-C chemokine receptor type 5 (CXCR5) and signaling lymphocyte activation molecule family member 6 (SLAMF6), and enriched genes of pathways associated with survival, memory and cytokine production (2, 5, 15). Tpex displayed stem-like properties and persisted without antigen stimulation (23–26). However, terminally exhausted subsets upregulated the expression of co-inhibitory receptors (PD1, TIM3, etc.) while lacking the expression of TCF-1 and CXCR5 (2). Terminally exhausted subsets enriched genes from pathways of apoptosis, cytotoxicity and cell division (5, 15). Therefore, terminally exhausted T cells mainly exert cytotoxic effects. Without treatment, terminally exhausted T cells would gradually become non-functional T cells and induce apoptosis (**Figure 1**) (6, 14, 15, 23). Current strategies to reinvigorate CD8^+^ TEXs are mainly by improving the function of these two subsets, which will be discussed in detail in the next chapter.

### 2.2 Massive inhibitory signals promote the progression of T cell exhaustion

CD8^+^ TEXs exhibited a “locked” dysfunctional state. Inhibitory signals (persistent antigen exposure, metabolic defeats, chronic interferon signaling, etc.) and exhaustion-related epigenetic modifications greatly altered gene transcription, contributing to the differentiation of CD8^+^ TEXs (1). These immunosuppressive factors are the important targets for the improvement of TEXs and cancer treatment.

#### 2.2.1 Persistent TCR signaling promotes the expression of exhaustion-related genes

**Figure 2:**
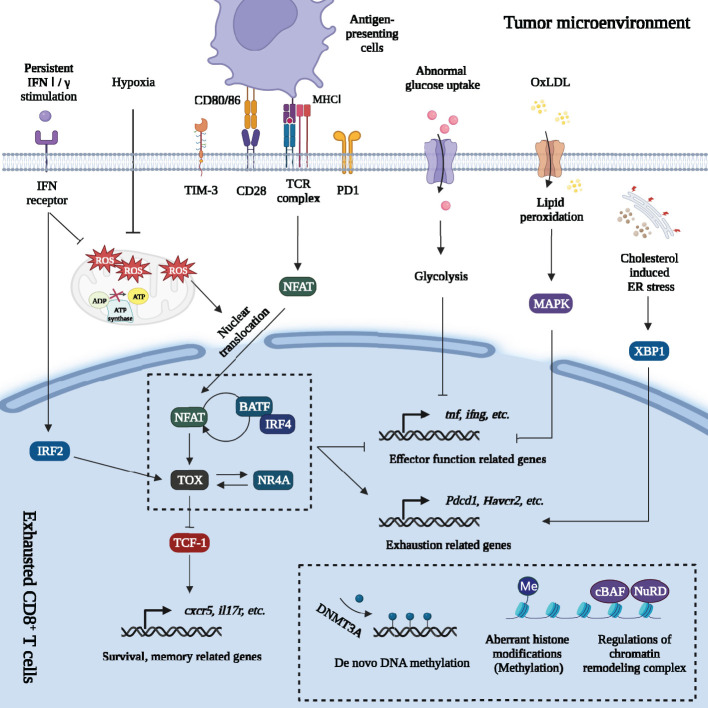
Regulations of gene expression of exhausted CD8^+^ T cells in tumor microenvironment. Massive signals in tumor microenvironment regulate gene expression of exhausted CD8^+^ T cells. **Firstly**, persistent TCR signaling promotes NFAT dephosphorylation and nuclear translocation. The decreased of AP-1 breaks the NFAT-AP-1 cooperation but drives the NFAT-dependent program. NFAT directly enhances PD1 and TIM3 expression and promotes exhaustion. NFAT activates other TFs including TOX and NR4A. Furthermore, NFAT forms a positive feedback loop with IRF4 and BATF. TOX directly regulates or cooperates with other TFs (IRF4: BATF dimer; NR4A, etc.) to increase the expression of exhaustion-related genes and downregulate expression of genes involved in inflammatory pathways. Besides, TOX inhibits the effects of TCF-1 and promotes the terminal exhaustion differentiation. **Secondly**, metabolic changes also affect gene expression. Abnormal glucose uptake in TEXs suppresses glycolysis, OXPHOS and damaged effector function. Excessive intake of OxLDL leads to lipid peroxidation, which activates MAPK and inhibits *Tnf* and *ifng* expression. Cholesterol increases ER stress and upregulates the expression of inhibitory receptors (PD1, 2B4) by activating ER stress sensor XBP1. Mitochondrial dysfunction and oxidative stress greatly promote terminal exhaustion program. The impaired OXPHOS and Blimp-1 related suppression of PGC1α-dependent mitochondrial reprogramming (under hypoxia and persistent antigen stimulation) greatly increased the level of mROS. mROS was a strong inducer of terminal exhaustion differentiation. **Thirdly,** epigenetic changes formed the “locked” dysfunctional state through *de novo* DNA methylation, aberrant histone methylation modification and regulation of chromatin remodeling complex on exhaustion-related genes. **Lastly**, persistent IFN signaling promoted terminal exhaustion progression by inducing aberrant lipid accumulation, elevating oxidative stress in CD8^+^ TILs and activating IRF2. Major Histocompatibility Complex (MHC I); Nuclear factor of activated T cells (NFAT); Activator protein-1 (AP-1); Programmed cell death protein 1 (PD1); T cell immunoglobulin domain and mucin domain-3 (TIM3); Thymocyte selection-associated high mobility group box protein (TOX); Nuclear receptor 4A (NR4A); Interferon regulatory factor 4 (IRF4); The basic leucine zipper activating transcription factor-like transcription factor (BATF); T cell factor-1 (TCF-1); Oxidative phosphorylation (OXPHOS); Oxidized low-density lipoprotein (OxLDL); p38 mitogen-activated protein kinase (MAPK); Endoplasmic reticulum (ER); X-box binding protein 1 (XBP1); Mitochondrial reactive oxygen species (mROS); Interferon (IFN); Interferon regulatory factor 2 (IRF2); DNA methyltransferases 3A (DNMT3A); canonical BRG1 or BRM-associated factor (cBAF); Nucleosome remodeling and deacetylase (NuRD).

The classical pathway of T cell exhaustion was driven by chronic TCR signaling, accompanied by a complex transcriptional network consisting of thymocyte selection-associated high mobility group box protein (TOX), nuclear factor of activated T cells 1 (NFATc1), nuclear receptor 4A (NR4A), T cell factor-1 (TCF-1) and activator protein-1 (AP-1) family transcription factors (TFs) (**Figure 2**) (27–30).

There were two main phases in T cell exhaustion: Tpex development phase and terminal exhausted differentiation phase (6, 15). During Tpex development phase, TCF-1, encoded by *tcf7*, mediated the transition from T-box expressed in T cells (T-bet) to eomesodermin (Eomes) and drove differentiation toward Tpex (15, 31). More importantly, TOX, the decisive TF of exhaustion, maintained Tpex through increasing the expression of *Tcf7, Bcl6, Bach2, Foxo1* and *Id3* and inhibiting the expression of *Il2ra* and *Irf4* (32). However, continuous inhibitory signals induced terminal exhausted differentiation. There were some significant changes of TFs in terminal exhausted differentiation phase, especially in the change of AP-1. AP-1 was a generic name, consisted of members of the Fos, Jun, Maf and ATF multigene families and was regulated by co-stimulatory signals (33). Usually, AP-1 (Jun-Fos dimer) interacted with NFATc1 and promoted T cell activation (29). However, most members of AP-1 family were dramatically downregulated in terminally exhausted CD8^+^ T cells in TME, excluding the basic leucine zipper activating transcription factor-like transcription factor (BATF). TFs including NFATc1, BATF, TOX, NR4A and interferon regulatory factor 4 (IRF4), greatly promote the development of terminal exhaustion. They were significantly increased in terminally exhausted subsets, resulting in increased expression of genes involved in exhaustion progression (*Havcr2, Tbx21, Ctla4, Pdcd1, Tigit*) and the decreased expression of genes of inflammatory pathways (*Ifng*, etc.) (22, 27, 29, 32, 34). Besides, in this phase, TOX inhibited the effects of TCF-1, promoting Tpex to differentiate into terminal exhausted subsets. TOX strongly drove terminal exhaustion differentiation (7, 30). Therefore, TEXs had high expression of multiple inhibitory receptors and limited cytotoxicity (**Figure 2**).

#### 2.2.2 Metabolic defeats regulate gene expression in exhausted CD8^+^ T cells

Metabolic defeats in CD8^+^ TEXs play a significant role to promote exhaustion differentiation. TME was an acidic environment with hypoxia, nutrient deficiency and high concentrations of metabolic waste, where TEXs underwent extensive metabolic reprogramming that promoted exhaustion-related gene expression (3, 35, 36).

In terms of nutrient metabolism, there was a decreased uptake of glucose but excessive accumulation of lipid and cholesterol in TEXs. CD8^+^ TEXs suffered from severe suppression of glycolysis and oxidative phosphorylation (OXPHOS) and increased lipid peroxidation and endoplasmic reticulum (ER) stress, which damaged the effector function of tumor infiltrating CD8^+^ T cells (CD8^+^ TILs) and promoted terminal exhaustion differentiation (**Figure 2**) (3, 36, 37).

Mitochondrial dysfunction and oxidative stress were of great importance to terminal exhaustion differentiation (20, 38, 39). CD8^+^ TEXs accumulated depolarized mitochondria with disrupted membrane, cristae structure and abnormal membrane potential (39). The impaired mitochondrial structures and functions significantly enhanced the level of mitochondrial reactive oxygen species (mROS) and oxidative stress (20, 38, 39). mROS was a strong inducer of terminal exhaustion differentiation. ROS activated the nuclear translocation of NFAT, boosting the transcription of TOX and exhaustion-related genes (20, 38). Furthermore, ROS activated phosphatase of activated cells 1 (PAC1). PAC1 downregulated the expression of genes including *Klrg1, Gzmb, Gzmm* by recruiting the nucleosome remodeling and deacetylase (NuRD) complex, which damaged effector function of CD8^+^ TEXs (**Figure 2**) (40).

#### 2.2.3 Epigenetic modifications promote the differentiation of exhausted CD8^+^ T cells

A broad remodeling of chromatin landscape was gradually established during the differentiation of CD8^+^ TEXs, including DNA methylation, histone modifications and chromatin remodeling in CD8^+^ TEXs. The alteration of chromatin accessibility regulated gene expression (41).

*De novo* DNA methylation, especially DNA methyltransferases 3A (DNMT3A)-dependent DNA methylation events, happened in some specific genes during the differentiation of TEXs. The *Ccr7* and *Tcf7* loci remained demethylated in Tpex (19, 42). However, in terminally exhausted T cells, genes involved in proliferation, tissue homing, metabolic activity and effector function (*Tcf7, Ccr7, Myc* and *ifng*) were acquired severe *de novo* DNA methylation, contributing to their low expression and terminal exhaustion differentiation (19, 42).

Aberrant histone modifications, particularly methylation modification, have been found in TEXs. The histone H3 lysine 27 (H3K27) demethylation of terminal-specific genes (*Tox*, etc.) and increased H3K27 methylation in progenitor-specific genes (*Tcf7*, etc.) contributed to the maintenance of terminally exhausted states (32). Besides, high levels of bivalent chromatin were found in terminally exhausted T cells, which was a phenomenon specific to TEXs in cancers. Bivalent chromatins were regulated by active H3K4me3 and repressive H3K27me3, poising genes for rapid expression or repression and responding to environmental stimulation (32). In hypoxia, bivalent genes were enriched in terminally exhausted subsets and suppressed the expression of genes of inflammatory response and leukocyte differentiation (32).

Genome-wide clustered regularly interspaced short palindromic repeats screens in mouse and human tumors indicated that chromatin remodeling complex, especially canonical BRG1 or BRM-associated factor (cBAF) was enriched in TEXs and downregulated effector function and the maintenance of exhaustion (43). Furthermore, ROS activated PAC1 and recruited chromatin remodeling complex NuRD complex, which suppressed the expression of genes involved in effector function (40).

In conclusion, massive inhibitory signaling in TME drove the progression of CD8^+^ T cell exhaustion. There are many strategies targeting on the inhibitory signals from TME or the key programs of exhaustion, showing the powerful anti-tumor effects in preclinical studies and some clinical trials (5, 18, 39, 42, 44). The reinvigoration of CD8^+^ TEXs in TME is a promising treatment to enhance the outcome of cancer patients.

## 3 Current strategies to reinvigorate exhausted CD8^+^ T cells in tumor microenvironment

**Figure 3:**
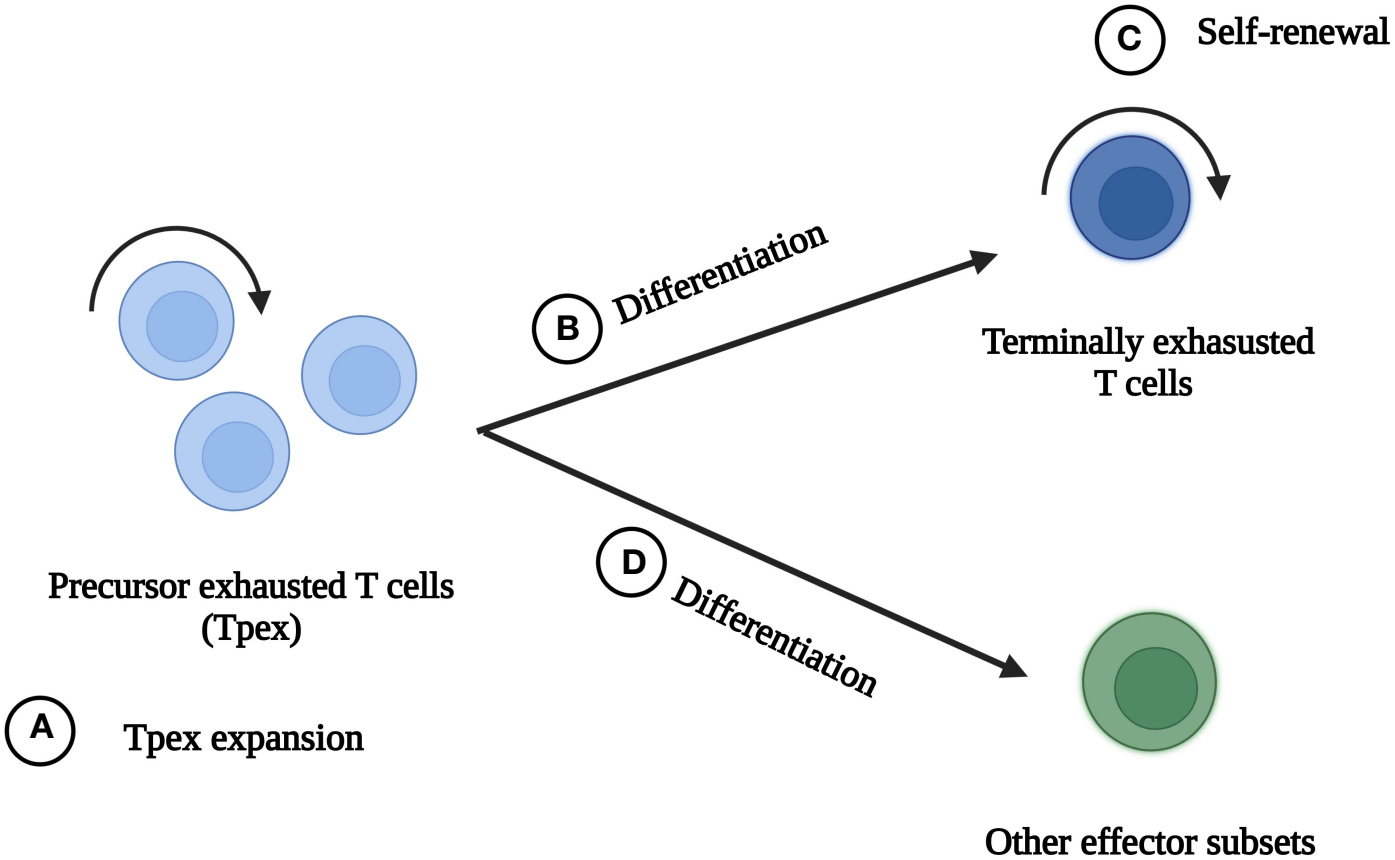
The main ideas to reinvigorate exhausted CD8^+^ T cells. There are four main ideas to reinvigorate exhausted CD8^+^ T cells in tumor microenvironment. **(A)** Tpex expansion and further expansion of terminally exhausted T cells. **(B)** Promotion of Tpex differentiation into terminally exhausted subsets with better effector function. **(C)** Self-renewal of terminally exhausted T cells. **(D)** Promotion of Tpex differentiation into other effector subsets.

Strategies including ICB, co-stimulatory receptor activation, transcription factor-based therapy, epigenetic therapy, metabolism-based therapy and cytokine therapy, have been reported to significantly improve CD8^+^ TEXs and boost anti-tumor immunity (5, 18, 39, 42, 44). Each of them has its own advantages and disadvantages. Until now, there is no way to completely reinvigorate exhausted state. These strategies can be divided into four types according to their main effects of improving TEXs (**Figure 3**): (A) To expand Tpex and further expand terminally exhausted T cells, of which ICB is one of the representatives. (B) To promote Tpex to differentiate into terminally exhausted subsets with superior cytotoxicity. Most of the strategies belong to this way. (C) To promote self-renewal of terminally exhausted T cells. Interleukin-10 (IL-10) therapy mainly played a role in this way. (D) To promote Tpex differentiate into other effector subsets, which can be achieved by transcription factor-based therapy and cytokine therapy. One treatment can improve TEXs through multiple ways. For example, ICB reinvigorated CD8^+^ TEXs by expanding Tpex and promoting them to differentiate into terminally exhausted subsets with better effector function.

We summarized the current strategies reported to reinvigorate TEXs to provide a basis for setting treatment strategies to reinvigorate TEXs, supress exhaustion progression in CAR-T cells and improve the prognosis in cancer patients.

### 3.1 Targeting coinhibitory or co-stimulatory receptor

**Figure 4:**
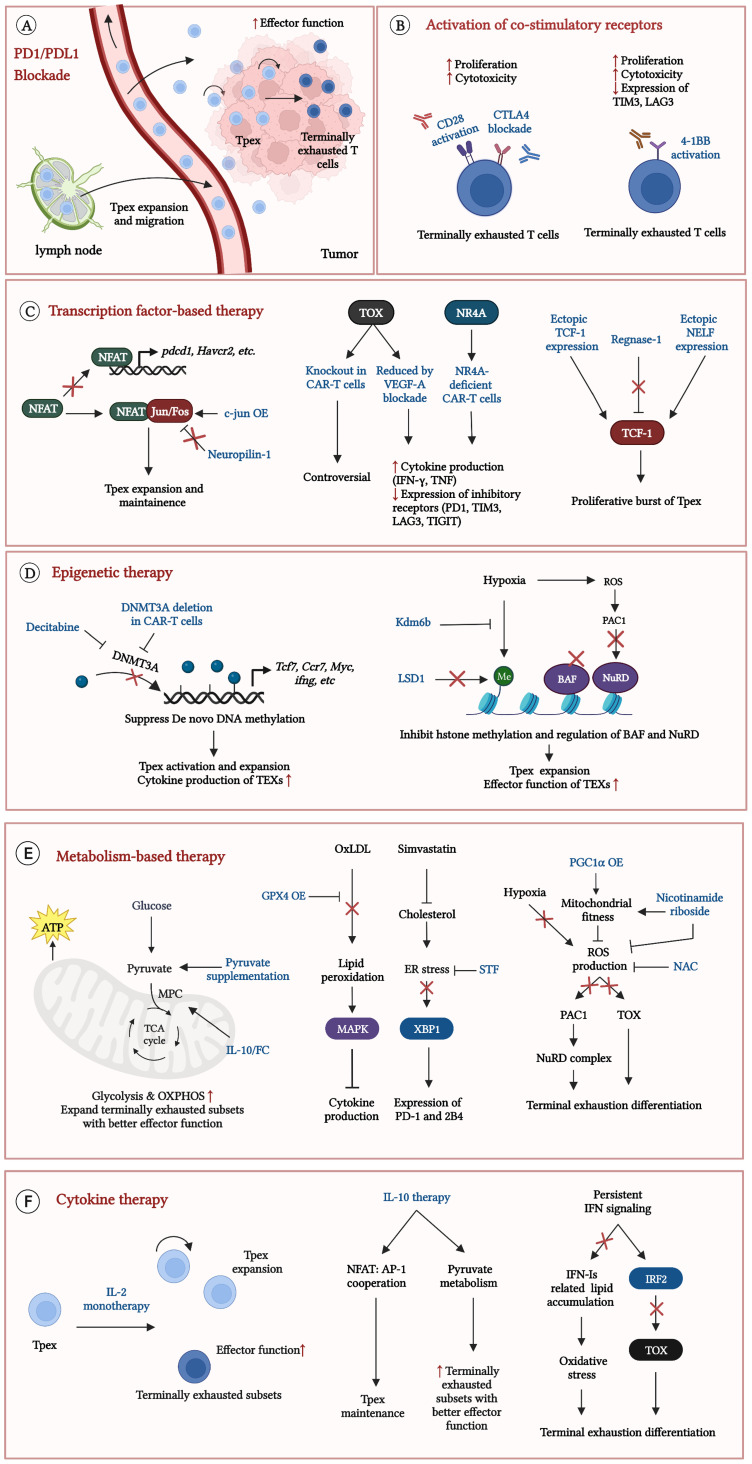
Current strategies to reinvigorate exhausted CD8^+^ T cells. Some strategies significantly reinvigorate exhausted CD8^+^ T cells in TME. **(A)** precursor exhausted T cells (Tpex) in lymph nodes expand, migrate to tumor and differentiate into terminally exhausted T cells with better effector function after PD1/PDL1 blockade; **(B)** Effects of activation CD28 or 4-1BB on TEXs; **(C)** Therapeutic efficacy in TEXs by regulations of exhaustion related transcription factors; **(D)** Epigenetic therapy results to the Tpex expansion and improves effector function in TEXs; **(E)** Improvement of metabolic states effectively reinvigorates TEXs; **(F)** Cytokine therapy expand Tpex and promote Tpex to differentiate into terminally exhausted T cells with better effector function. Exhausted T cells (TEXs); Programmed cell death protein 1 (PD1); Programmed cell death ligand 1 (PDL1); Cytotoxic T-lymphocyte-associated protein 4 (CTLA4); Chimeric antigen receptor (CAR); T cell immunoglobulin domain and mucin domain-3 (TIM3); Lymphocyte activation gene-3 (LAG3); T cell immunoreceptor with immunoglobulin and ITIM domain (TIGIT); Nuclear factor of activated T cells (NFAT); CAR-T cells overexpressing c-Jun (c-Jun OE); type I interferons (IFN-Is); Interferon (IFN); Interferon regulatory factor 2 (IRF2); Interleukin-10 (IL-10); Thymocyte selection-associated high mobility group box protein (TOX); Nuclear receptor 4A (NR4A); Vascular endothelial growth factor-A (VEGF-A); T cell factor-1 (TCF-1); Negative elongation factor (NELF); DNA methyltransferases 3A (DNMT3A); Lysine-specific demethylase 6B (Kdm6b); Lysine specific demethylase1 (LSD1); canonical BRG1 or BRM-associated factor (cBAF); Nucleosome remodeling and deacetylase (NuRD); Reactive oxygen species (ROS); Phosphatase of activated cells 1 (PAC1); Mitochondrial pyruvate carrier (MPC); Oxidative phosphorylation (OXPHOS); Oxidized low-density lipoprotein (OxLDL); Glutathione peroxidase 4 (GPX4); p38 mitogen-activated protein kinase (MAPK); Endoplasmic reticulum (ER); X-box binding protein l (XBP1); Peroxisome proliferator-activated receptor-γ coactivator 1 α (PGC1α); N-acetylcysteine (NAC); Interleukin-2 receptor (IL2-R).

The common feature of TEXs was the enhanced expression of multiple co-inhibitory receptors (PD1, TIM3, LAG3 etc.) and downregulation of CD28, resulting in the lack of co-stimulatory signals but massive inhibitory signals (1, 5). Blockade of co-inhibitory receptors, especially PD1/programmed cell death ligand 1 (PDL1) blockade, showed outstanding efficacy to reinvigorate TEXs (5, 15, 23). PD1/PDL1 blockade is the main strategy for T cell exhaustion. In addition, activation of co-stimulatory receptor, particularly 4-1BB, has been considered a promising therapeutic approach in recent years (**Figure 4**) (18, 32, 45–47).

#### 3.1.1 PD1/PDL1 blockade

PD1/PDL1 blockade is the important component of tumor immunotherapy. Various studies have suggested that Tpex are the main responders to PD1/PDL1 blockade (5, 15, 23). The understanding of the effects of PD1/PDL1 blockade on CD8^+^ TEXs is constantly being updated. Initially, it was believed that PD1/PDL1 blockade could reverse CD8^+^ TEXs (2). However, further research revealed that the development of TEXs was accompanied by complex transcriptional and epigenetic programs and it was difficult to reverse the TEXs (2, 5). Current research suggested that the main effects of PD1/PDL1 blockade were to expand Tpex in TME. PD1/PDL1 blockade induced the replenishment of Tpex from outside the tumor, especially from lymph nodes, bridging systemic and anti-tumor responses (23–26, 48, 49). However, the expansion of Tpex was partly based on local expansion of pre-existing Tpex (25, 26, 49). Besides, PD1/PDL1 blockade promoted Tpex to differentiate into terminally exhausted subsets with better effector function (**Figure 4A**) (23–26, 48, 49). Anti-PD1 therapy improved the gene expression of inflammatory pathways, resulting in better effector function (5, 14, 15). However, there were minimal changes in epigenetic profiles were observed in CD8^+^ TEXs, suggesting that PD1/PDL1 blockade failed to change their epigenetic status (5, 32). CD8^+^ T cells inevitably became dysfunctional after treatment because the key exhaustion programs did not change. This may explain the poor outcome or failure to set up durable responses in some cancer patients with anti-PD1 therapy.

In preclinical models, anti-PD-1 therapy significantly suppressed the growth of murine melanoma, resulting in the proliferative burst of Tpex and increased the number of terminally exhausted CD8^+^ T cells with superior cytotoxicity (**Table 1**) (5). Furthermore, the expansion of Tpex after treatment was related to better outcome of cancer patients (5, 23). A clinical study evaluating the efficacy of anti-PD1 therapy (Nivolumab) in patients with advanced melanoma showed that the increased frequency of Tpex was associated with the prolonged progression-free survival and overall survival in responders (5). Another study showed that in patients of non-small-cell lung cancer (NSCLC) treated with anti-PD1 therapy (Pembrolizumab), responsive tumors showed an expansion of Tpex with low expression of co-inhibitory molecules and high expression of GZMK, while non-responsive tumors failed to accumulate Tpex (23). In clinical practice, PD1/PDL1 blockade showed its powerful effects on many cancers including melanoma, liver cancer, etc. But its low response rate and drug resistance limited its application (16, 17). There are a large number of clinical trials evaluating the efficacy of anti-PD1 antibody monotherapy or combination therapy on solid tumors while few of them evaluate the change in TEXs. Improving CD8^+^ TEXs may be a breakthrough point to reverse the current dilemma of cancer immunotherapy. More studies are needed to explore the improvement of TEXs after anti-PD1 therapy in human solid tumors.

**Table 1:** The main preclinical studies targeting coinhibitory or co-stimulatory receptors.

Target	Effects	Tumor type	Species	Combined therapy	Ref
**PD1**	**Anti PD-1 therapy** 1. In vivo; 2. Inhibited tumor growth; 3. Expanded Tpex and promoted Tpex differentiate into terminally exhausted cells with better effector function;	Melanoma (B16-OVA)	M	/	(5)
	**Anti PD-1 therapy** 1. In vivo; 2. Expanded Tpex and promoted Tpex differentiate into terminally exhausted cells;	Melanoma (B16)	M	/	(15)
**CTLA-4**	**Anti CTLA-4 antibody** 1. In vivo; 2. Prolonged survival; 3. Expanded CD8^+^ TILs and increased IFN-γ production;	Ovarian cancer (OVCAR5)	M	PD1/PDL1 blockade	(46)
**4-1BB**	**4-1BB agonistic antibody** 1. In vivo; 2. limited tumor progression; 3 .Improved the metabolic sufficiency of CD8^+^ TILs;	Melanoma (B16-F10)	M	PD1/PDL1 blockade	(46)
	**4-1BB agonistic antibody** 1. In vivo; 2. Prolonged survival; 3. Increased numbers of CD8^+^ TILs with greater IFN-γ production and the decreased expression of PD-1, TIM3, and LAG3;	Glioblastoma (CT2A)	M	PD1/PDL1 blockade	(50)
	**4-1BB agonistic antibody** 1. Ex vivo (co-cultured drug with CD8^+^ TILs from patients) 2. Promoted proliferative capacity and cytokine production;	Liver cancer	H	PD1/PDL1 blockade	(18)
	**4-1BB agonistic antibody** 1. Ex vivo (co-cultured drug with CD8^+^ TILs from patients); 2. Enhanced proliferation and production of IFN-γ and TNF-α in CD8^+^ TILs;	Ovarian cancer	H	PD1/PDL1 blockade	(51)
	**4-1BB-CD3zeta CAR-T cells** 1. ACT (transfer of 4-1BB-CD3zeta CAR-T cells into tumor models); 2. Exerted persistent anti-tumor activity; 3. 4-1BB-CD3zeta CAR-T cells expressed genes involved in T-cell memory program;	Lymphoma (Raji)	M	/	(52)

The table summarized the main preclinical studies of targeting coinhibitory or co-stimulatory receptors. Tumor, tumor cell lines in mouse model or method. H, human, M, mouse; Ref, references; ACT, adoptive cell transfer therapy.

#### 3.1.2 Activation of co-stimulatory receptor

CD28 superfamily and tumor necrosis factor receptor (TNFR) superfamily were the major components of co-stimulatory receptors (53). CD8^+^ TEXs showed significantly impaired co-stimulatory signals which inhibited NFAT: AP-1 cooperation and induced metabolic dysfunction, resulting to the progression of terminal exhaustion (32, 46). Activation of CD28 and 4-1BB (a member of TNFR superfamily) has been demonstrated their therapeutic potential in some studies (18, 46, 47, 50, 51).

##### 3.1.2.1 CD28 stimulation

CD28 was significantly downregulated in CD8^+^ TEXs (32). CD28 signaling can be improved by direct or indirect stimulation. CD28 agonistic antibody raised severe side effects (extensive T-cell activation) in early clinical trials, making it hard to directly activate CD28 on TEXs (54). Recently, tumor-specific antigen (TSA) x CD28 bispecific antibody brought hope to direct CD28 stimulation. TSA x CD28 bispecific antibody showed limited toxicity and powerful effects when combined with TSA x CD3 bispecific antibody (54). Further research was needed to evaluate the effects of the TSA x CD28 bispecific antibody on TEXs.

Cytotoxic T-lymphocyte-associated protein 4 (CTLA4) blockade was an alternative choice to improve CD28 signaling. CTLA-4 greatly inhibited CD28 signaling. CTLA4 was highly expressed on TEXs and inhibited the binding between CD28 and CD80/CD86. Besides, CTLA4 downregulated the expression of CD28 on TEXs (45, 47). In mouse models of ovarian cancer, CTLA4 blockade effectively prolonged survival and expanded CD8^+^ TILs with increased IFN-γ production. Furthermore, CTLA4 blockade increased the effects of anti-PD1 therapy and this combination therapy enhanced the proliferative capacity, cytotoxicity and expression of T-Bet in TEXs (**Figure 4B**) (**Table 1**) (47). However, CD28 expression was critical for the efficacy of CD28 stimulation and CTLA4 blockade (47). How to restore or upregulate CD28 expression on TEXs may be another important issue.

##### 3.1.2.2 4-1BB stimulation

4-1BB provides co-stimulatory signals independent of CD28 and has excellent therapeutic potential (53). Activation of 4-1BB reinvigorated TEXs in several ways. Direct activation of 4-1BB enhanced the expression of AP-1 family members (*Atf3*, *Batf3* and *Mafb*) and restored the expression of key inflammatory genes (32). Besides, it induced metabolic reprogramming by increasing mitochondrial fusion and biogenesis, which significantly improved mitochondrial function and energy supply (**Figure 4B**) (46).

4-1BB was mainly expressed on terminally exhausted subsets of several cancers including liver cancer, ovarian cancer and glioma (18, 46, 51). 4-1BB signaling can be improved by agonistic antibody or CAR-T cells construction. In preclinical models, 4-1BB agonistic antibody monotherapy effectively limited the growth of murine melanoma and glioblastoma (46, 50). 4-1BB agonistic antibody greatly increased effector function of terminally exhausted subsets, which showed increased interferon-γ (IFN-γ) and tumor necrosis factor alpha (TNF-α) production (18, 50, 51). More importantly, the combination of 4-1BB agonistic antibody and anti-PD1 therapy contributed to the better control of cancers and the further promotion of proliferative function, cytotoxicity and downregulation expression of TIM3 and LAG3 in TEXs (**Table 1**) (18, 46, 50, 51). A phase Ib clinical trial (NCT02179918) evaluated the anti-tumor activity of 4-1BB agonistic antibody in combination with PD-1-blocking mAb (Pembrolizumab) in patients with advanced solid tumors. It showed the efficacy and safety of this combination (55). 26.1% of the patients had confirmed complete or partial responses and there were no dose-limiting toxicities. Compared to non-responders, responders showed a higher levels of activated memory/effector CD8^+^ T cells in peripheral blood (55). In addition, 4-1BB was a practical choice to construct CAR-T cells. 4-1BB-CD3zeta CAR-T cells expressed genes involved in T-cell memory program and showed persistent anti-tumor activity in mouse lymphoma models, while CD28-CD3zeta CAR-T cells failed to produce long-term effects (52).

In total, 4-1BB is an ideal target of immunotherapy with high specificity and powerful effect. The efficacy of 4-1BB therapy depended on the expression level of 4-1BB (50). Therefore, individualized treatment was required. Furthermore, 4-1BB agonistic antibody led to the loss of CD266, which limited the efficacy of ICB. Preservation of CD226 expression was important when combining 4-1BB activation with anti-PD1 therapy (56).

### 3.2 Transcription factor-based therapy

T cell exhaustion has unique transcriptional features and transcription factor-based therapy can precisely regulate the expression of key TFs, resulting in the altered expression of exhaustion-related genes expression. The main targets of transcription factor-based therapy including NFAT: AP-1 cooperation, TOX, NR4A, TCF-1, BATF and IRF4, which can be regulated by drugs or CAR-T cells therapy.

#### 3.2.1 NFAT: AP-1 cooperation

NFAT: AP-1 (Jun/Fos complex) cooperation was significantly damaged in CD8^+^ TEXs. Upregulation of c-Jun expression by drug or CAR-T cell therapy effectively stabilized NFAT: AP-1 cooperation, which inhibited the NFAT-dependent exhaustion program, expanded Tpex and greatly enhanced anti-tumor immunity (12, 57, 58).

In preclinical models, transfer of c-Jun OE (CAR-T cells overexpressing c-Jun) significantly limited the growth of murine leukemia and osteosarcoma. C-Jun OE showed enhanced expansion potential, cytotoxicity and were resistant to terminal exhaustion differentiation (12). Besides, neuropilin-1 (NRP1) is an inhibitory receptor on CD8^+^ T cells. NRP1 limited the Tpex self-renewal by suppressing c-jun expression. In mouse melanoma models, knockdown *Nrp1* in CD8^+^ T cells increased the frequency of Tpex and memory T cells in TME. *Nrp1* ^–/–^ T cells exhibited greater effector function upon re-stimulation, promoting long-term immunity (57). More importantly, it improved the efficacy of ICB, resulting in the enhanced tumor clearance (**Figure 4C**) (**Table 2**) (57). In short, targeting c-Jun effectively suppressed the development of terminal exhaustion in several cancers.

**Table 2:** The main preclinical studies of transcription factor-based therapy.

Target	Effects	Tumor type	Species	Combined therapy	Ref
**NFAT: AP-1 complex**	**c-Jun OE (**CAR-T cells overexpressing c-Jun**)** 1. ACT (Transfer of c-Jun OE into tumor models) 2. Limited tumor growth; 3. c-Jun OE showed enhanced expansion potential, cytotoxicity and diminished terminal exhaustion differentiation;	Leukemia (Nalm6-GD2); Osteosarcoma (143B)	M	/	(12)
	**Knockdown *Nrp1* in CD8^+^ T cells** 1. Evaluated the anti-tumor effects of Knockdown Nrp1 in CD8^+^ T cells; 2. Increased the frequency of Tpex and memory T cells in TME; 3. Exhibited greater effector function upon re-stimulation and promoted long-term immunity;	Melanoma (B16)	M	PD1/PDL1 blockade	(57)
**TOX**	**Tox DKO (TOX1, TOX2) CAR-T cells** 1. ACT (transfer of Tox DKO CAR-T cells into tumor models); 2. Limited tumor growth; 3. Promoted cytokine production (TNF, IFN-γ); 4. Downregulated the expression of inhibitory receptors (PD-1, TIM3 and LAG3);	Melanoma (B16)	M	/	(30)
	**TOX-knockout tumor-specific T cells** 1. ACT (transfer of TOX-knockout tumor-specific T cells into tumor models); 2. TOX-knockout tumor-specific T cells showed: 3. Non-exhausted phenotype; 4. Loss of effector function; 5. Increased apoptosis;	Melanoma (B16)	M	/	(22)
	**Anti-VEGFR2 antibody** 1. In vivo; 2. Suppressed tumor growth and prolonged survival; 3. Decreased the level of TOX; 4. Reduced the expression of inhibitory receptors (TIM3, LAG3 and TIGIT); 5. Increased IFN-γ and TNF production;	Colon cancer (MC38-OVA)	M	PD1/PDL1 blockade	(59)
**NR4A**	**NR4A-deficient CAR-T cells** 1. ACT (transfer of NR4A-deficient CAR-T cells into tumor models) 2. Suppressed tumor growth; 3. Promoted cytokine production (TNF, IFN-γ); 4. Downregulated the expression of inhibitory receptors;	Melanoma (B16)	M	/	(30)
**TCF1**	**Ectopic TCF-1 expression** 1. Evaluated the anti-tumor effects of ectopic TCF-1 expression; 2. Suppressed tumor growth; 3. Significant expansion of Tpex and terminally exhausted subsets; 4. Enhanced cytokine production and decreased expression of co-inhibitory receptors in terminally exhausted subsets;	Melanoma (B16)	M	/	(44)
	**Regnase-1-deficient CAR-T cells** 1. ACT (transfer of Regnase-1-deficient CAR-T cells into tumor models); 2. Enhanced tumor clearance; 3. CAR-T cells showed the features of Tpex and long-term persistence in TME;	ALL (h CD19^+^ B-ALL cells)	M	/	(60)
	**Ectopic NELF expression** 1. Evaluated the anti-tumor effects of ectopic NELF expression; 2. Boosted anti-tumor immunity; 3. Improved the proliferative and cytotoxic function of CD8^+^ TILs;	Breast cancer (E0771, AT3)	M	/	(61)

The table summarized the main preclinical studies of transcription factor-based therapy. Tumor: tumor cell lines in mouse model or method. M, mouse; Ref, references; ACT, adoptive cell transfer therapy.

#### 3.2.2 TOX and NR4A

TOX and NR4A were significantly increased in terminally exhausted subsets and profoundly promote the transcriptional program of T cell exhaustion (30). Targeting TOX is controversial. *Seo et al. (2019)* described that Tox DKO (TOX1, TOX2) CAR-T cells slowed down the progression of mouse melanoma. Tox DKO (TOX1, TOX2) CAR-T cells in TME showed significantly increased cytokine production (TNF, IFN-γ) and decreased expression of inhibitory receptors ((PD-1, TIM3, and LAG3) (30). However, *Scott et al. (2019)* provided a contrary conclusion. Although they displayed non-exhausted phenotype (low expression of inhibitory receptors), TOX-knockout T cells remained dysfunctional and showed increased apoptosis in mouse melanoma (22). TOX-knockout T cells failed to persist in TME. TOX induced T cell exhaustion might prevent chronic activation-induced cell death. Further research was needed to investigate the status of TOX-knockout T cells in TME. However, the decreased expression of TOX resisted the development of exhaustion. Anti-vascular endothelial growth factor-A (VEGFA) antibody was reported to greatly limit tumor growth and reduce the levels of TOX and NFAT in mouse colon cancer models. Anti-VEGFA antibody decreased expression of inhibitory receptors (PD1, TIM3, LAG3, TIGIT), and increased IFN-γ and TNF production in CD8^+^ TEXs (59). More importantly, it achieved better anti-tumor effects when combined with PD1/PDL1 blockade. However, NR4A-deficient CAR-T cells showed similar effects of Tox DKO CAR-T cells (**Figure 4C**) (**Table 2**) (30, 62). NR4A knockout may be a safe choice when TOX has complex effects on T cell fate.

#### 3.2.3 TCF-1

TCF-1 is essential for the differentiation of memory and exhausted T cells (14, 15). Increasing the expression of TCF-1 led to a proliferative burst of Tpex (44, 60, 61). Ectopic TCF-1 expression in preclinical tumor models significantly suppressed tumor growth and expanded Tpex and terminally exhausted subsets. Furthermore, terminally exhausted subsets enhanced cytokine production (IL2, TNF, IFN-γ) and decreased expression of co-inhibitory receptors (PD1, 2B4, LAG3) (44). Besides, Regnase-1 and Negative Elongation Factor (NELF) were reported to regulate *tcf7* expression in TEXs (60, 61). The ribonuclease Regnase-1 was the upstream negative regulator of TCF-1. Regnase-1 targeted *Tcf7* messenger RNA and suppressed the formation of Tpex. Regnase-1-deficient CAR-T cells enhanced the clearance of murine acute lymphoblastic leukemia (ALL). It showed the features of Tpex and the long-term persistence in TME (60). Besides, NELF, an RNA polymerase II pausing factor, cooperated with TCF-1 to enhance the expression of TCF-1 target genes. Ectopic NELF expression boosted anti-tumor immunity in mouse model of mammary tumor and improved the proliferative and cytotoxic function of CD8^+^ TILs (**Figure 4C**) (**Table 2**) (61). In short, enhanced expression of TCF-1 resulted in a better anti-tumor response.

#### 3.2.4 BATF and IRF4

BATF and IRF4 were significantly upregulated in TEXs where BATF combined with IRF4 to promote terminal exhausted differentiation (29, 32). BATF regulated both effector programs and exhausted differentiation (63). The way to engineer BATF in CAR-T cells remained controversial. *Seo et al. (2021)* showed that overexpression of BATF on CAR-T cells reduced exhaustion program and increased anti-tumor immunity in murine melanoma. Depletion of BATF decreased the number of T cells and poor tumor control (64). However, *Zhang et al. (2022)* presented that the depletion of BATF in CAR-T cells increased the expression of TCF-1 and IL-7 receptor alpha and suppressed expression of genes involved in terminal exhaustion differentiation, leading to the better anti-tumor responses in murine melanoma (65).

The contradictory effects of IRF4 were similar to that of BATF. Both IRF4 knockdown and overexpression in CAR-T cells were reported to increase cytokine production (64, 65). The difference may be associated with differences in the construction of CAR-T cells and the type of tumor models, etc. Therefore, further research was needed to investigate the role of BATF and IRF4 in TME.

In conclusion, transcription factor-based therapy made it possible to exert precise regulation of gene expression, contributing to the Tpex expansion and TEXs with better effector function. However, TFs usually had extensive effects and led to different outcomes. The overall effects of TFs need to be clarified before the treatment. Most of the strategies mentioned above remain in the preclinical stage. Further studies are needed to evaluate their function in clinical practice.

### 3.3 Epigenetic therapy

Epigenetic changes toward key genes of T cell exhaustion have gradually occurred during the exhaustion differentiation, mainly including *de novo* DNA methylation (especially DNMT3A-dependent DNA methylation), histone modification and regulation of chromatin remodeling complexes (41). Epigenetic therapy greatly improved exhaustion by removing modifications obtained during the process of exhaustion.

#### 3.3.1 Suppressing *de novo* DNA methylation

The *De novo* DNA methylation, especially DNA methyltransferases 3A (DNMT3A)-dependent DNA methylation events in some specific genes promoted the progression of terminal exhaustion (19, 42). Application of DNA demethylating agents Decitabine (DAC) or DNMT3A knockout CAR-T cells effectively inhibited the *de novo* DNA methylation of exhaustion-related genes (13, 42).

In preclinical studies, pre-treatment of DAC prior to PDL1 blockade significantly inhibited the growth of murine prostate cancer, resulting in CD8^+^ T cells resisting exhaustion and maintaining greater expansion potential (42). More importantly, DAC had a synergistic effect with anti-PD1 antibody, promoting to a further Tpex expansion and cytokine production (IFN-γ, TNF) of CD8^+^ TILs in several mouse tumor models (19). Furthermore, DNMT3A knockout CAR-T cell therapy effectively raised anti-tumor responses. In murine breast cancer and glioma models, DNMT3A knockout CAR-T cells significantly suppressed tumor growth and prolonged survival. They enhanced IL-10 production and differentiated into stem-like CAR-T cells, maintaining long-term anti-tumor responses (**Figure 4D**) (**Table 3**) (13).

**Table 3:** The main preclinical studies of epigenetic therapy.

Target/ Process	Effects	Tumor type	Species	Combined therapy	Ref
**DNA methylation**	**Decitabine (DAC)** 1. In vivo; 2. Mainly evaluated the efficacy when combined with anti-PD1 therapy; 3. Combined therapy inhibited tumor growth; 4. CD8^+^ TILs resisted exhaustion and maintained greater expansion potential;	Prostate Cancer (TRAMP-C2)	M	PD1/PDL1 blockade	(42)
	**Decitabine (DAC)** 1. In vivo; 2. Mainly evaluated the efficacy when combined with anti-PD1 therapy; 3. Combined therapy promoted Tpex expansion; 4. Increased secretion of IFN-γ and TNF-α; 5. Potentiated CD8^+^ T cell response;	Colon cancer (MC38); T cell Lymphoma (EG7)	M	PD1/PDL1 blockade	(19)
**DNMT3A**	**DNMT3A knockout CAR-T cells** 1. ACT (transfer of DNMT3A knockout CAR-T cells into tumor models); 2. Slowed down tumor growth and prolonged survival; 3. Differentiated into stem-like CAR-T cells in TME; 4. Maintained long-term anti-tumor responses;	Breast cancer (LM7); Glioma (U373)	M	/	(13)
**Kdm6b**	**Overexpressing Kdm6b on T cells** 1. ACT (transfer of Kdm6b overexpressing on T cells into tumor models); 2. Limited tumor growth; 3. Achieved better cytotoxicity; 4. No effects on the differentiation program to exhaustion;	Melanoma (B16)	M	/	(32)
**LSD1**	**LSD1 blockade** 1. In vivo; 2. Limited tumor growth; 3. Mainly expanded Tpex;	Colon cancer (MC38)	M	PD1/PDL1 blockade	(66)
	**LSD1 inhibitor** 1. In vivo; 2. Inhibited tumor progression; 3. Increased CD8^+^ T cell infiltration and proliferative capacity;	Breast cancer (EMT6; 4T1)	M	PD1/PDL1 blockade	(67)
	**nLSD1 blockade** 1. In vivo 2. Increased CD8^+^ T cell infiltration, particularly the IFN-γ^+^ CD8^+^ T cells; 3. Decreased the expression of TIGIT, LAG3 and TIM3 on CD8^+^ TILs;	Breast cancer (4T1 TNBC; MDA-MB-231 TNBC)	M	PD1/PDL1 blockade	(68)
**Arid1a**	**Arid1a-deficient CAR-T cells** 1. ACT (transfer of Arid1a-deficient CAR-T cells into tumor models); 2. Prevented acquirement of exhaustion-related chromatin accessibility; 3. Drove the differentiation into memory T cells;	Colon cancer (MC38)	M	/	(43)
	**Arid1a inhibitor** 1.ACT (tansfer of Naive CD8^+^ T cells pre-treat with Arid1a inhibitor); 2. Suppressed tumor growth; 3. CAR-T cells showed greater persistence and better anti-tumor activity;	Melanoma (B16); Colon cancer (MC38)	M	/	(69)
**PAC1**	**Knockdown *pac1*** 1. Evaluated the anti-tumor effects of knockdown of *pac1*; 2. Limited tumor growth; 3. Increased CD8^+^ TILs; 4. Increased production of IFN-γ and TNF in CD8^+^ TILs; 5. Potentiated CD8^+^ T cell response;	Colon cancer (AOM-DSS model); Melanoma (B16-F10)	M	/	(40)

The table summarized the main preclinical studies of epigenetic therapy. Tumor: tumor cell lines in mouse model or method. M, mouse; Ref, references; ACT, adoptive cell transfer therapy.

#### 3.3.2 Improving histone methylation modifications

Aberrant histone modification occurred during the progression of terminal exhaustion differentiation, particularly methylation modification, which was closely related to hypoxia in TME. Targeting the histone demethylase lysine-specific demethylase 6B (Kdm6b) and lysine specific demethylase1 (LSD1) showed great therapeutic potential (32, 66–68). Kdm6b, a less oxygen-sensitive member of the KDM6 family of H3K27 demethylases, was downregulated in terminally exhausted subsets. Transfer of T cells overexpressing Kdm6b significantly limited the growth of mouse melanoma. Kdm6b overexpression improved the effector function of T cells but had no effect on the key differentiation program of TEXs (**Figure 4D**) (**Table 3**) (32).

LSD1 significantly reinvigorated CD8^+^ TEXs. LSD1 specifically removed both mono- and di-methylation of histone H3K4 and H3K9 and mediated immunosuppressive effects independent of histone modifications. LSD1 was significantly upregulated in CD8^+^ T cells in many cancers, which was correlated with prognosis of cancer patients (66–68). In preclinical studies, LSD1 blockade significantly suppressed the progression of colon and breast cancer in mice (66, 67). LSD1 blockade significantly enhanced the amount of intra-tumoral Tpex by promoting the *tcf7* transcriptional activity (66). Furthermore, LSD1 and PD1/PDL1 blockade displayed cooperative effects. The combination of LSD1 inhibitor and anti-PD1 therapy achieved a further expansion of Tpex and promoted Tpex differentiate into terminally exhausted CD8^+^ T cells with better cytotoxicity (66, 67). However, the efficacy of LSD1 blockade depended on antigenicity of cancer cells. LSD1 blockade may be useless in low antigenicity tumors, requiring additional antigen stimulation (66). There is a difference in the efficacy of targeting the LSD1 nucleus (nLSD1) or the LSD1. Phosphorylated nLSD1 induced nuclear entry and retention of EOMES, contributing to T cell exhaustion. nLSD1 blockade increased the infiltration of CD8^+^ T cells, particularly the IFN-γ^+^ CD8^+^ T cells in murine breast cancer. Besides, it decreased the expression of TIGIT, LAG3 and TIM3 of CD8^+^ TILs (**Figure 4D**) (**Table 3**) (68). In conclusion, LSD1 can regulate TCF-1 and EOMES and is a promising therapeutic target to improve TEXs. There are some ongoing clinical trials evaluating the efficacy of LSD1 inhibitor in advanced solid tumors (NCT03895684, NCT05268666, etc.).

#### 3.3.3 Targeting chromatin remodeling complexes

Chromatin remodeling complexes (cBAF, NuRD, etc.) promoted the expression of genes involved in exhaustion differentiation (40, 43). Ablating cBAF complex subunit Arid1a in CAR-T cells prevented acquirement of exhaustion-related chromatin accessibility, and drove the differentiation into memory T cells in TME (43). Besides, pre-treatment of naive CD8^+^ T cells with Arid1a inhibitor markedly promoted their effector function and increased anti-tumor efficacy in mouse melanoma and colon cancer models (69). Notably, Arid1a blockade was suitable for pre-treatment of CAR-T cells. The use of Arid1a inhibitor *in vivo* decreased the effects of immunotherapy, which might be due to the broad regulatory effect of chromatin remodeling complex (**Figure 4D**) (**Table 3**) (69, 70).

Mitochondrial dysfunction and oxidative stress greatly increased the level of mROS. ROS-PAC1-NuRD complex downregulated the expression of genes including *Klrg1, Gzmb, Gzmm*, thereby damaging the effector function of TEXs. Knockdown of *pac1* effectively suppressed the growth of murine melanoma and colon cancer. Moreover, it increased the number CD8^+^ TILs, significantly improved effector function of TEXs and increased anti-tumor immunity (**Figure 4D**) (**Table 3**) (40). In conclusion, therapeutic strategies based on epigenetic regulation can effectively inhibit differentiation toward terminal TEXs, but exert a broader range of effects. The safety of treatment must be taken seriously. The same target may have different effects with different interventions.

### 3.4 Metabolism-based therapy

CD8^+^ TEXs suffered from severe metabolic defects in TME (3, 20, 38, 71). There was a close relationship between T-cell exhaustion and metabolic signals. Reducing metabolic stress can effectively reinvigorate TEXs and activate anti-tumor responses.

#### 3.4.1 Improving of pyruvate metabolism

Severe suppression of glycolysis and OXPHOS was a feature of TEXs, which severely damaged effector function and promoted the terminal exhaustion differentiation. It can be improved by pyruvate supplementation and regulations of pyruvate metabolism (3, 71–73).

Supplementation of pyruvate enhanced both glycolysis and OXPHOS, resulting in improved effector function of CD8^+^ TILs in murine melanoma (71). The improvement of pyruvate transportation has showed excellent therapeutic efficacy. Pyruvate is transported into the mitochondria by the mitochondrial pyruvate carrier (MPC) (72). *Guo, et al. (2021)* described that the half-life-extended Interleukin-10-Fc fusion protein (IL-10/Fc) can promote MPC to transport pyruvate into mitochondria, increasing OXPHOS and mitochondrial function. Surprisingly, IL-10/Fc treatment effectively controlled the growth of murine melanoma. It promoted self-renewal of terminally exhausted subsets and improved their proliferative capacity and cytotoxicity (72). IL-10/Fc treatment significantly improved the efficacy of PD1/PDL1 blockade, which induced a durable efficacy (**Figure 4E**) (**Table 4**) (72). IL-10/Fc treatment plays a unique role in the improvement of TEXs. Further research is needed to evaluate its effects in clinical practice.

**Table 4:** The main preclinical studies of metabolism-based therapy.

Process	Effects	Tumor type	Species	Combined therapy	Ref
**Glycolysis**	**Pyruvate** 1. Ex vivo (co-cultured exogenous pyruvate and CD8^+^ TILs); 2. Enhanced both glycolysis and OXPHOS; 3. Improved cytokine production (IFN-γ, TNF-α);	Melanoma (B16)	M	/	(71)
**Pyruvate metabolism**	**IL-10 Fc** 1. In vivo; 2. Limited tumor growth; 3. Expanded terminally exhausted subsets (self-renewal); 4. Improved cytotoxicity of terminally exhausted subsets;	Melanoma (B16); Colon cancer (CT26)	M	PD1/PDL1 blockade	(72)
**Lipid peroxidation**	**GPX4 OE (CAR-T cells overexpressing GPX4)** 1. ACT (transfer of GPX4 OE into tumor models); 2. Enhanced tumor control; 3. Increased the number of CD8^+^ TILs; 4. GPX4 OE showed enhanced cytokine production (TNF, IFN-γ);	Melanoma (B16); Colon cancer (MC38)	M	/	(36)
**ER stress**	**ER-stress inhibitor STF or simvastatin** 1. ACT (transfer of CD8^+^ T cells pre-treatment with drugs); 2. Enhanced tumor control; 3. Decreased the expression of PD1 and 2B4;	Melanoma (B16); Colon cancer (MC38)	M	/	(37)
**Mitochondrial metabolism**	**Nicotinamide ribose** 1. Oral treatment; 2. Better control of tumor; 3. Increased cytokine production (IFN, TNF);	Melanoma (YUMM1.7)	M	PD1/PDL1 blockade	(39)
	**N- acetylcysteine (NAC)** 1. ACT (transfer of T cells pre-treatment with NAC); 2. Suppressed tumor growth; 3. Increased effector function (Granzyme B, TNF, IFN);	Melanoma (B16)	M	PD1/PDL1 blockade	(38)
	**Knockdown *pac1*** 1. Evaluated the anti-tumor effects of knockdown of *pac1*; 2. Limited tumor growth; 3. Increased CD8^+^ TILs; 4. Increased production of IFN-γ and TNF in CD8^+^ TILs; 5. Potentiated CD8^+^ T cell response;	Colon cancer (AOM-DSS model); Melanoma (B16-F10)	M	/	(40)
	**PGC1αOE (CAR-T cells overexpressing PGC1α)** 1. ACT (transfer of CAR-T cells overexpressing PGC1α into tumor model); 2. Limited tumor growth; 3. PGC1αOE without progenitor exhausted signature; 4. Altered differentiation and avoided the differentiation of TEXs;	Melanoma (B16);	M	/	(20)
**Hypoxia**	**Metformin** 1. In vivo 2. Monotherapy is not effective; 3. Increased the effects of PD1/PDL1 blockade;	Melanoma (B16); Colon cancer (MC38)	M	PD1/PDL1 blockade	(74)
	**Axitinib (tyrosine kinase inhibitor)** 1. In vivo; 2. Decreased tumor burden and improved survival; 3. Decreased expression of PD1 and TIM3; 4. Increased production of IFN and TNF;	Melanoma (B16);	M	PD1/PDL1 blockade	(20)

The table summarized the main preclinical studies of metabolism-based therapy. Tumor: tumor cell lines in mouse model or method. M, mouse; Ref, references; ACT, adoptive cell transfer therapy.

#### 3.4.2 Releasing lipid peroxidation and endoplasmic reticulum stress

CD8^+^ TEXs in TME accumulated high levels of oxidized low-density lipoprotein (OxLDL) and cholesterol, leading to the severe lipid peroxidation and ER stress. The reduction of lipid peroxidation and ER stress helped to prevent exhaustion progression (36, 37).

Glutathione peroxidase 4 (GPX4) can decrease the lipid peroxidation induced by OxLDL. Transfer of CAR-T cells overexpressing GPX4 (GPX4 OE) significantly suppressed the growth of murine melanoma and colon cancer. GPX4 OE showed an increase of cytokine production, boosting effector function in TEXs (36). Furthermore, the inhibition of ER stress led to a low expression of inhibitory receptors. Transfer T cells pre-treatment with ER-stress inhibitor STF or cholesterol-lowering drug simvastatin significantly slowed down the growth of mouse melanoma and colon cancer. Furthermore, pre-treatment decreased their apoptosis and expression of PD1 and 2B4, contributing to the enhanced anti-tumor immunity (**Figure 4E**) (**Table 4**) (37).

#### 3.4.3 Reducing mitochondrial oxidative stress and hypoxia

Reducing mitochondrial oxidative stress and remodeling mitochondrial metabolism inhibited differentiation into terminally exhausted subsets (20, 38, 39). ROS was a strong inducer of terminally exhausted differentiation. Reducing ROS production or inhibiting its downstream exerted therapeutic effects in several preclinical studies (20, 38, 39).

The supplementation of Nicotinamide riboside improved mitochondrial fitness and decreased ROS production, contributing to the increased cytokine production and a better control of melanoma in mice (39). In addition, N- acetylcysteine (NAC), the antioxidant that neutralizes ROS, can decrease the levels of ROS and TOX expression in TEXs, thereby limiting terminal exhausted differentiation (20, 38). Transfer of T cells pre-treated with NAC effectively suppressed tumor growth in the mouse melanoma model. The pre-treatment increased the effector function of CD8^+^ T cells. More importantly, it increased the effects of PD1/PDL1 blockade and improved the survival of tumor-bearing mice (38). Besides, the knockdown of PAC1, the downstream of ROS, promoted proliferative and cytotoxic functions of TEXs and considerably slowed tumor growth (40). Lastly, CAR-T cells overexpressing PGC1α (PGC1αOE) showed excellent therapeutic potential. PGC1αOE suppressed the growth of murine melanoma. Surprisingly, PGC1αOE did not show any progenitor exhausted signature. Overexpression of PGC1α altered the differentiation of CD8^+^ T cells and prevented the differentiation of TEXs. PGC1α is a promising target to prevent the exhaustion of CAR-T cells (**Figure 4E**) (**Table 4**) (20).

Hypoxia promoted terminal exhaustion and decreased the sensitivity of immunotherapy (20, 32, 74, 75). Normalizing hypoxia in TME is an important way to improve the response to immunotherapy. Metformin (drug treatment for type 2 diabetes) can effectively reduce hypoxia in TME and inhibit oxygen consumption in tumor cells. It also improved the efficacy of PD1 blockade (74). In addition, low doses of the tyrosine kinase inhibitor Axitinib (targeting vascular endothelial growth factor receptors) improved the tortuous vasculature and decreased hypoxia in TME. Axitinib significantly decreased tumor burden and improved survival. It reduced the expression of PD1 and TIM3 and promoted the production of IFN and TNF, suppressing the terminal exhaustion differentiation. Besides, Axitinib cooperated with PD1 or CTLA4 blockade to reduce tumor burden and improve survival (**Figure 4E**) (**Table 4**) (20).

Therefore, metabolism is deeply coordinated with immune signaling pathway, influencing differentiation of tumor-infiltrating CD8^+^ T cells. Metabolism-based therapy prevents CD8^+^ T cells from differentiating into terminal exhaustion and improves efficacy of ICB and CAR-T cell therapy.

### 3.5 Cytokine therapy

Cytokine therapy has been widely used in anti-tumor treatment (4). Cytokines, such as IL-2, IL-10, TGF-β and IFN, play a complex role in the regulation of immunity, which can promote or suppress anti-tumor immunity in different situations. We mainly focus on their main effects in TME. It was reported that the lack of IL-2 and IL-10 signaling and chronic IFN stimulation induced the terminal exhaustion progression (58, 72, 76, 77). Cytokine therapy acts an important role in reinvigorating TEXs.

#### 3.5.1 IL-2

IL-2, the potent multifunctional cytokine, mainly activated effector and memory T cells and enhanced anti-tumor immunity (78). The production of IL-2 was severely damaged in CD8^+^ TEXs. Although it was reported that continuous high levels of IL-2 in TME led to T cell exhaustion by activating aryl hydrocarbon receptors (79). More studies indicated that the IL-2 therapy significantly activated TEXs and enhanced anti-tumor responses, especially when combined with PD1/PDL1 blockade (21, 80, 81).

IL-2 agonists such as IL-2/NARA1 complex, SIL2-mesenchymal stem cells (MSCs) have been demonstrated to reinvigorate TEXs in murine melanoma and colon cancer models. IL-2 monotherapy significantly suppressed tumor growth and prolonged survival. It significantly expanded tumor-specific CD8^+^ T cells with better effector function and low expression of PD1, TIM3 and LAG3 (80). In addition, IL-2 therapy expanded Tpex and promoted the production of IFN-γ of terminal exhausted T cells (81). More importantly, IL-2 signaling closely correlated with the efficacy of PD1/PDL1 blockade (21, 81). Combined IL-2 therapy with PD1/PDL1 blockade showed more powerful anti-tumor responses (21, 81). This combination therapy greatly inhibited tumor growth and overcame ICB resistance of murine melanoma and colon cancer (81). In addition, PD-1-cis-targeted IL-2Rβγ agonists, can bind to PD-1 and IL-2Rβγ and activate the two molecules. PD-1-cis-targeted IL-2Rβγ agonists led to large expansion of CD8^+^ TILs in murine pancreatic tumor models. Surprisingly, the new effector CD8^+^ T cells showed the low expression of exhaustion-related genes such as *Havcr2*, *Tigit* and *Tox* but the increased expression of genes involved in productive and protective immune memory. In contrast to IL-2 or anti-PD1 monotherapy, the combination of anti-PD1 therapy and IL-2 activation promoted Tpex to differentiate into better effector subsets, rather than terminally exhausted CD8^+^ T cells with better effector function (21). PD-1-cis-targeted IL-2Rβγ agonists showed better anti-tumor efficacy than pembrolizumab in treating the tumor model of human PD-1-transgenic mice (**Figure 4F**) (**Table 5**) (21). In conclusion, IL-2 therapy is a promising therapy for the improvement of TEXs, especially with the combination of PD1/PDL1 blockade.

**Table 5:** The main preclinical studies of cytokine therapy.

Target	Effects	Tumor type	Species	Combined therapy	Ref
**IL-2**	**IL-2/NARA1 complex** 1. IL-2 and NARA1 (NARA1, high-affinity CD25 mimic, avoiding vascular leak syndrome caused by Tregs activation); 2. In vivo; 3. Inhibited tumor growth; 4. Expanded tumor-specific CD8^+^ T cells with better effector function;	Melanoma (B16-F10)	M	/	(80)
	**SIL2-EMSCs** 1. Engineered MSCs for targeting IL-2 to activate CD8 T cells in TME; 2. In vivo; 3. Expanded Tpex; 4. Promoted the production of IFN-γ in terminal exhausted T cells;	Melanoma (B16); Colon cancer (MC38, CT26)	M	PD1/PDL1 blockade	(81)
	**PD-1-cis-targeted IL-2Rβγ agonists** 1. Bound to PD-1 and IL-2Rβγ; 2. In vivo; 3. Suppressed tumor growth; 4. Expanded CD8^+^ TILs with better effector function; 5. Decreased the expression of exhaustion-related genes such as *Havcr2*, *Tigit* and *Tox* in CD8^+^ TILs; 6. Increased expression of genes involved in productive and protective immune memory in CD8^+^ TILs;	Pancreatic tumor (Panc02-H7)	M	/	(21)
**IL-10**	**IL-10/Fc** 1. In vivo; 2. Expanded terminally exhausted subsets (self-renewal); 3. Improved cytotoxicity of terminally exhausted subsets;	Melanoma (B16-F10); Colon Cancer (CT26)	M	PD1/PDL1 blockade	(72)
**IFN**	**Knockdown *irnar1* in T cells** 1. Evaluated the anti-tumor effects of knockdown of *irnar1*; 2. Inhibited the growth; 3. Increased the number of CD8^+^ TILs, characterized by the decreased expression of TIM3, CTLA-4, LAG3 and TOX and the enhanced expression of TCF1;	Liver cancer (Delivering oncogenes NRAS and AKT into hepatocytes)	M	PD1/PDL1 blockade	(82)
	**IRF2-deficient CD8^+^ T cells (CD8-*irf2* cKO mice)** 1. ACT (transfer of IRF2-deficient CD8^+^ T cells into tumor model); 2. Slowed down tumor growth; 3. Low expression of inhibitory receptors and the enhanced production of Granzyme B, perforin and IFN-γ; 4. No expression of TOX;	Colon Cancer (MC38)	M	PD1/PDL1 blockade	(76)

The table summarized the main preclinical studies of cytokine therapy. Tumor: tumor cell lines in mouse model or method. M, mouse; Ref, references; ACT, adoptive cell transfer therapy.

#### 3.5.2 IL-10

IL-10 therapy effectively reinvigorated TEXs by promoting NFAT: AP-1 cooperation and improving pyruvate metabolism, which were mentioned above (58, 72). IL-10 signaling inhibited the activation-induced exhaustion of CD8 T cells in TME. IL-10 signaling blockade significantly impaired the maintenance of Tpex in the mouse model of chronic lymphocytic leukemia (CLL) and accelerated the development of exhaustion (58). More importantly, IL-10 therapy showed the powerful effects in improving terminally exhausted subsets. In murine melanoma models, IL-10/Fc treatment promoted their self-renewal and improved the effector function by upregulating mitochondrial pyruvate carrier-dependent oxidative phosphorylation (72). In addition, the combination of IL-10/Fc and PD1/PDL1 blockade showed stronger anti-tumor efficacy, contributing to a durable efficacy in the mouse CT26 colorectal tumor model (**Figure 4F**) (**Table 5**) (72).

A phase 1b clinical trial (NCT 02009449) evaluated the efficacy of PEGylated IL-10 with anti-PD-1 monoclonal antibody (Pembrolizumab or Nivolumab) in patients with advanced solid tumors (83–85). PEGylated IL-10 monotherapy induced objective tumor responses in patients with renal cell cancer (RCC). Besides, it significantly increased the number of CD8^+^ TILs, especially the LAG3^+^ PD1^+^ CD8^+^ T cells and enhanced the production of IFN-γ and Granzyme B (83, 84). The combination therapies showed positive outcome in previously treated patients with RCC and NSCLC with objective responses of 43% and 40%, respectively. However, the change in CD8^+^ T cells was not evaluated (85). Therefore, IL-10 therapy has great potential in reinvigorating CD8^+^ TEXs and boosting anti-tumor immunity. And more research is needed to evaluate the efficacy and effects on CD8^+^ TILs of the combination of IL-10 therapy and PD1/PDL1 blockade in patients with solid tumors.

#### 3.5.3 IFN

The stimulation of type I interferons (IFN-Is) and IFN-γ played a significant role in the activation of immunity. However, persistent interferon stimulation in TME drove the terminal exhaustion process of CD8^+^ T cells and severely limited anti-tumor responses (76, 77). CD8^+^ TEX enriched for IFN-I-stimulated genes. IFN-Is induced the aberrant lipid accumulation and elevated oxidative stress in CD8^+^ TILs, which triggered mitochondrial defects and induced terminal exhaustion process (82). Besides, persistent IFN-Is and IFN-γ signaling activated the key TF interferon regulatory factor 2 (IRF2). IRF2 regulated genes involved in IFN signaling, TNFα/NF-κB signaling and immune exhaustion (*tox*), suppressing the effects of CD8^+^ T cells in TME (76).

Inhibiting the persistent IFN signaling from TME significantly prevented the development of T cell exhaustion in preclinical models (76, 82). Knockdown of the expression of *ifnar1*(encoding the receptor of IFN-Is) in T cells significantly inhibited the growth of murine liver cancer. There was an increased number of CD8^+^ TILs, characterized by the decreased expression of TIM3, CTLA-4, LAG3 and TOX and the enhanced expression of TCF1 (82). Besides, knockdown of *irf2* on CD8^+^ T cells showed powerful efficacy. Transfer of IRF2-deficient CD8^+^ T cells significantly limited the growth of murine colon cancer. IRF2-deficient CD8^+^ T cells showed low expression of inhibitory receptors and the enhanced production of Granzyme B, perforin and IFN-γ (76). More importantly, the effects of immunosuppression from persistent IFN-Is and IFN-γ siganling were inhibited in IRF2-deficient CD8^+^ T cells, resulting to long-term tumor control. Surprisingly, there was almost no TOX expression in IRF2-deficient CD8^+^ T cells. IRF2 depletion prevented the acquisition of T cell exhaustion program during persistent IFN signaling in TME (76). IRF2-deficient CD8^+^ T cells are the strong candidates for CAR-T cell therapies (**Figure 4F**) (**Table 5**) (76).

Besides, blockade of IFN signaling enhanced the anti-tumor effects of PD1/PDL1 blockade (76, 82). The level of IFN-I-stimulated genes was related to the poor efficacy of ICB in patients of melanoma or breast cancer, and anti-IFNAR-1 antibody overcame the drug resistance of anti-PD1 antibody in mouse liver cancer (82). Furthermore, the combination of IRF2-deficient CD8^+^ T cells significantly with anti-PDL1 antibody significantly led to the durable tumor control in the mouse model of breast cancer (76). However, there are no clinical applications to target IFN signaling (IFNI, IRF2) in TME. In short, IFNI and IRF2 are the promising targets for CAR T-cell construction and further investigation is needed.

## 4 Tips for applying strategies to improve exhausted CD8^+^ T cells

Some strategies have been found to play an important role in improving CD8^+^ TEXs (5, 18, 39, 42, 44). Each of these strategies had its own advantages and disadvantages. We discussed the tips for applying strategies to improve CD8^+^ TEXs, especially on the efficacy and safety of treatments and combination therapy.

### 4.1 Efficacy and safety of treatments

The specificity and safety of the target must be considered when determining a treatment strategy. Different targets have different preferences for intervention. Drug therapy and CAR-T cell therapy targeting on the same molecule may lead to opposite outcomes. For example, Arid1a-deficient CAR-T cell therapy markedly suppressed tumor growth in mouse colon cancer models while the use of Arid1a inhibitor *in vivo* decreased anti-tumor efficacy (43, 69, 70). Besides, there was a slight difference in the role and goal of drug therapy and CAR-T cell therapy. Drug therapies exert extensive effects *in vivo*, leading to the improvement of existing TEXs and inhibitory signals in TME. ICB and metabolism-based therapy are the representatives. In contrast, CAR-T cell therapy can specifically overexpress or knockdown the key gene on T cells and mainly improve the T cells that were transferred into the body. The design of CAR-T cell therapy should take into account the suppression of exhaustion progression where targets including 4-1BB, DNMT3A and Arid1a have showed their superiority (13, 43, 52). It’s worth comparing the effects of drugs and CAR-T cells in preclinical studies against the same target.

Safety is the foundation of effective treatment strategies. It’s important to consider how to improve the precision of the treatment to limit side effects, especially when targeting molecules that directly activate immunity (TFs, cytokines, agonistic antibodies; demethylating agents). For example, CD28 agonistic antibodies led to extensive T-cell activation and a severe cytokine storm in early clinical trials. The use of TSA x CD28 bispecific antibody enhanced artificial synapse between T cell and its target cell, avoiding the side effects of extensive CD28 activation (54). It is essential to completely evaluate the safety of treatment strategies in preclinical studies. By increasing target specificity, we can decrease the toxicity of treatment and achieve precision treatment.

When applying strategies to improve CD8^+^ TEXs, the features of therapeutic targets should be considered. Cancers are highly heterogeneous, and different patients respond differently to treatment strategies. It was reported that the frequency of Tpex correlated with better efficacy of PD1/PDL1 blockade. The non-responsive tumors failed to accumulate Tpex in lung cancer (23). The therapeutic efficacy and response time of ICB may be limited in patients with poor Tpex infiltration (5). For patients with poor Tpex infiltration, strategies targeting on terminally exhausted subsets (4-1BB activation, IL-10 therapy, etc.) may be a good choice (18, 72). Besides, preclinical studies demonstrated that the efficacy of 4-1BB therapy depended on the expression level of 4-1BB (50). Also, the efficacy of LSD1 blockade was closely associated with antigenicity of cancer cells. LSD1 blockade may be useless in tumors with low antigenicity, requiring additional antigen stimulation (66). Therefore, pre-treatment assessment of the critical factors helps to choose the effective treatment strategy.

However, the majority of current strategies are still at the preclinical stage and were shown to improve TEXs in several murine tumor models. To promote the translation of theoretical mechanisms into clinical applications, complete studies are needed to evaluate their efficacy in more tumor models. Furthermore, researchers should optimize drug design to increase treatment efficacy and reduce side effects. Lastly, preclinical drug safety evaluation and clinical trials are needed to conducted to evaluate the efficacy and safety of treatment in cancer patients.

### 4.2 Combination therapy

ICB, especially PD1/PDL1 blockade, is the important component of cancer immunotherapy and the most studied strategy to improve TEXs. PD1/PDL1 blockade showed outstanding efficacy to reinvigorate TEXs (5, 15, 23). Most of the studies on combination therapy focused on PD1/PDL1 blockade-based combination therapy. We mainly summarized the current combination strategies and predicted other promising combination therapies.

**Figure 5:**
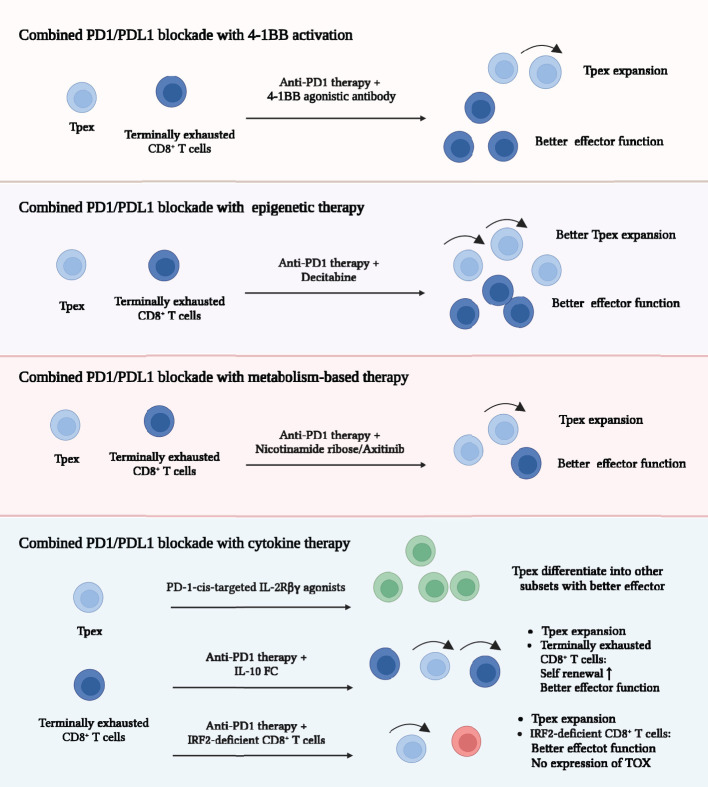
Combined strategies for improving exhausted CD8^+^ T cells. Strategies including 4-BB activation, epigenetic therapy, metabolism-based therapy and cytokine therapy cooperate with PD1/PDL1 blockade and markedly increase anti-tumor immunity. Combination of 4-BB activation and PD1/PDL1 blockade promotes greater number of terminally exhausted subsets with improved cytotoxicity. Combination of epigenetic therapy and PD1/PDL1 blockade leads to a further Tpex expansion and enhanced effector function of TEXs. Combination of metabolism-based therapy and PD1/PDL1 blockade further increases the cytotoxicity of terminally exhausted subsets. Combination of cytokine therapy and PD1/PDL1 blockade effectively enhances anti-tumor response in different ways. Programmed cell death protein 1 (PD1); Programmed cell death ligand 1 (PDL1); Interleukin-10-Fc fusion protein (IL-10/Fc); Interferon regulatory factor 2 (IRF2); Thymocyte selection-associated high mobility group box protein (TOX).

Anti-PD1 or anti-PDL1 monotherapy can effectively recruit Tpex from lymph nodes and promote the differentiation of terminally exhausted subsets with better effector function (5, 15, 23). However, it has a severe limitation that CD8^+^ T cells inevitably became dysfunctional because key programs of exhaustion did not change (5, 32, 42). Therefore, it seems that the optimal solution is to combine different strategies to complement deficiencies and achieve maximum exhaustion suppression. Treatments including 4-BB activation, epigenetic therapy, metabolism-based therapy and cytokine therapy have been reported to cooperate with ICB and markedly increase anti-tumor immunity (**Figure 5**) (5, 18, 39, 42). Firstly, combination of PD1/PDL1 blockade and 4-1BB activation is powerful. 4-1BB was mainly expressed on terminally exhausted subsets and 4-1BB activation significantly improved their effector function. This combination therapy achieved a large number of terminally exhausted subsets with better cytotoxicity, which was demonstrated to achieve significant tumor control (18, 32, 47, 50, 51). Secondly, epigenetic therapy can remove epigenetic modification obtained during the exhaustion differentiation, which complements deficiencies of ICB. When combined with ICB, epigenetic therapy led to a further Tpex expansion and enhanced effector function (32, 42, 66, 67, 69, 70). Thirdly, metabolism-based therapy contributed to metabolic reprogramming and most of them decreased terminal exhaustion differentiation (20, 38, 39, 58, 73). It was of great importance to improve the immunosuppressive environment because the exhaustion program was constantly developed without the elimination of inhibitory signals. Metabolism-based therapy is comparatively safe and significantly improves the efficacy of ICB. Lastly, cytokine therapy exerted unique effects when combining with PD1/PDL1 blockade. IL-2 therapy may modify the exhaustion differentiation of CD8^+^ T cells. The combination of anti-PD1 therapy and IL-2 activation promoted Tpex to differentiate into better effector subsets, rather than terminally exhausted CD8^+^ T cells (21). The combination of IL-10 Fc with anti-PD1 therapy greatly expanded the terminally exhausted subsets with improved the effector function (72). In summary, combination therapies were reported to achieve a better tumor control, which may be the superior choice for cancer patients.

Furthermore, we predicted some promising choices for combination therapy. Overexpression of 4-1BB or depletion of DNMT3A and Arid1a in CAR-T cells have shown the great effects in suppressing the acquirement of exhaustion program in CAR-T cells (13, 43, 52). The combination of PD1/PDL1 blockade and CAR-T cells mentioned above may trigger superior anti-tumor effects. Anti-PD1 therapy significantly expanded the existing Tpex and promoted them differentiate into terminal exhausted subsets with better effector function (5, 15, 23). CAR-T cells can provide an additional source of cytotoxic cells. More importantly, these CAR-T cells did not acquire exhaustion program, contributing to the long-term anti-tumor responses. In addition, the combination of metabolism-based therapy with epigenetic therapy seemed to be a promising choice. Hypoxia is a strong inducer of the acquirement of exhaustion-related epigenetic regulation (32). Improvement of mitochondrial metabolism effectively reduced hypoxia and suppressed terminal exhausted differentiation (38–40). This combination therapy may raise a further suppression of acquiring exhaustion-related epigenetic regulation, contributing to the powerful anti-tumor response. Further research is needed to explore the efficacy of the above combination therapy.

In conclusion, when applying strategies to improve TEXs, physicians and researchers should focus on the specificity and safety of each strategy and perform individualized treatment. The combination therapy showed superior efficacy in suppressing tumor growth. More studies are needed to evaluate the long-term effects of different combination strategies.

## 5 Discussion

CD8^+^ TEXs maintained a persistent dysfunctional state, regulated by complex transcriptional, epigenetic programs and profound metabolic reprogramming (1, 3, 5). T cell exhaustion was probably an adaptive change to prevent overstimulation and activation-induced cell death in TME, but it severely limited anti-tumor immunity and the efficacy of CAR-T cell therapy (9–13, 22). As the main responder to PD1/PDL1 blockade, TEXs may play a significant role in reversing the current dilemma of cancer immunotherapy (5, 14, 15, 22). Strong therapeutic potential has been observed when combining ICB with other therapies including 4-1BB activation, metabolism-based therapy, epigenetic therapy and cytokine therapy (18, 32, 42, 59, 70, 72, 77). Furthermore, optimization of CAR-T cells modifications suppressed the terminal exhaustion differentiation, especially the engineered CAR-T cells with overexpression or knockdown of TFs (13, 20, 60, 76). However, there were a lot of unknowns, especially the determining program driving the differentiation toward memory or exhausted subsets, and the possibility of inducing Tpex to differentiate into a better effector subset and even block the entire exhausted program. Understanding these mechanisms will allow new horizons in the fate decision of exhaustion and reveal the potential therapeutic targets. The ideal goal of CD8^+^ TEXs reinvigoration is to boost anti-tumor immunity as much as possible, without causing immune-related tissue damage, prolong survival, suppress terminal exhaustion differentiation and contribute to the achievement of tumor elimination and better clinical outcomes in cancer patients.

## Author contributions

DX: Conceptualization (lead); Writing-review and editing (lead). QTG: Conceptualization (equal); writing-original draft (lead); Writing-review and editing (lead). MH: Writing-original draft (supporting); Writing-review and editing (equal). QHG: Writing -review and editing (equal). FY: Writing-review and editing (supporting). MW: Writing-review and editing (supporting). QN: Writing-review and editing (supporting). All authors contributed to the article and approved the submitted version.

## References

[1] Philip M Schietinger A. CD8(+) T cell differentiation and dysfunction in cancer. Nat Rev Immunol (2022) 22(4):209–23. doi: 10.1038/s41577-021-00574-3 PMC9792152 34253904

[2] Kallies A Zehn D Utzschneider DT. Precursor exhausted T cells: key to successful immunotherapy? Nat Rev Immunol (2020) 20(2):128–36. doi: 10.1038/s41577-019-0223-7 31591533

[3] Reina-Campos M Scharping NE Goldrath AW. CD8(+) T cell metabolism in infection and cancer. Nat Rev Immunol (2021) 21(11):718–38. doi: 10.1038/s41577-021-00537-8 PMC8806153 33981085

[4] Propper DJ Balkwill FR. Harnessing cytokines and chemokines for cancer therapy. Nat Rev Clin Oncol (2022) 19(4):237–53. doi: 10.1038/s41571-021-00588-9 34997230

[5] Miller BC Sen DR Al Abosy R Bi K Virkud YV LaFleur MW. Subsets of exhausted CD8(+) T cells differentially mediate tumor control and respond to checkpoint blockade. Nat Immunol (2019) 20(3):326–36. doi: 10.1038/s41590-019-0312-6 PMC6673650 30778252

[6] Beltra JC Manne S Abdel-Hakeem MS Kurachi M Giles JR Chen Z. Developmental relationships of four exhausted CD8(+) T cell subsets reveals underlying transcriptional and epigenetic landscape control mechanisms. Immunity (2020) 52(5):825–41. doi: 10.1016/j.immuni.2020.04.014 PMC8360766 32396847

[7] Khan O Giles JR McDonald S Manne S Ngiow SF Patel KP. TOX transcriptionally and epigenetically programs CD8(+) T cell exhaustion. Nature (2019) 571(7764):211–8. doi: 10.1038/s41586-019-1325-x PMC6713202 31207603

[8] Jansen CS Prokhnevska N Master VA Sanda MG Carlisle JW Bilen MA. An intra-tumoral niche maintains and differentiates stem-like CD8 T cells. Nature (2019) 576(7787):465–70. doi: 10.1038/s41586-019-1836-5 PMC7108171 31827286

[9] Barsch M Salie H Schlaak AE Zhang Z Hess M Mayer LS. T-Cell exhaustion and residency dynamics inform clinical outcomes in hepatocellular carcinoma. J Hepatol (2022) 77(2):397–409. doi: 10.1016/j.jhep.2022.02.032 35367533

[10] Jiaqiang BH Z Goswami S Meng L DD Z CM C. PD1(Hi) CD8(+) T cells correlate with exhausted signature and poor clinical outcome in hepatocellular carcinoma. J Immunother Cancer. (2019) 7(1):331. doi: 10.1186/s40425-019-0814-7 31783783 PMC6884778

[11] Thommen DS Koelzer VH Herzig P Roller A Trefny M Dimeloe S. A transcriptionally and functionally distinct PD-1(+) CD8(+) T cell pool with predictive potential in non-small-cell lung cancer treated with PD-1 blockade. Nat Med (2018) 24(7):994–1004. doi: 10.1038/s41591-018-0057-z 29892065 PMC6110381

[12] Lynn RC Weber EW Sotillo E Gennert D Xu P Good Z. C-jun overexpression in CAR T cells induces exhaustion resistance. Nature (2019) 576(7786):293–300. doi: 10.1038/s41586-019-1805-z 31802004 PMC6944329

[13] Prinzing B Zebley CC Petersen CT Fan YP Anido AA Yi ZZ. Deleting DNMT3A in CAR T cells prevents exhaustion and enhances antitumor activity. Sci Transl Med (2021) 13(620):eabh0272. doi: 10.1126/scitranslmed.abh0272 34788079 PMC8733956

[14] Kurtulus S Madi A Escobar G Klapholz M Nyman J Christian E. Checkpoint blockade immunotherapy induces dynamic changes in PD-1(-)CD8(+) tumor-infiltrating T cells. Immunity (2019) 50(1):181–194.e6. doi: 10.1016/j.immuni.2018.11.014 30635236 PMC6336113

[15] Siddiqui I Schaeuble K Chennupati V Marraco SAF Calderon-Copete S Ferreira DP. Intratumoral Tcf1(+)PD-1(+)CD8(+) T cells with stem-like properties promote tumor control in response to vaccination and checkpoint blockade immunotherapy. Immunity (2019) 50(1):195–211.e10. doi: 10.1016/j.immuni.2018.12.021 30635237

[16] Chow A Perica K Klebanoff CA Wolchok JD. Clinical implications of T cell exhaustion for cancer immunotherapy. Nat Rev Clin Oncol (2022) 19(12):775–90. doi: 10.1038/s41571-022-00689-z PMC10984554 36216928

[17] Bagchi S Yuan R Engleman EG. Immune checkpoint inhibitors for the treatment of cancer: clinical impact and mechanisms of response and resistance. Annu Rev Pathol (2021) 16:223–49. doi: 10.1146/annurev-pathol-042020-042741 33197221

[18] Kim HD Park S Jeong S Lee YJ Lee H Kim CG. 4-1BB delineates distinct activation status of exhausted tumor-infiltrating CD8(+) T cells in hepatocellular carcinoma. Hepatology (2020) 71(3):955–71. doi: 10.1002/hep.30881 PMC7154753 31353502

[19] Li X Li Y Dong L Chang Y Zhang X Wang C. Decitabine-priming increases anti-PD-1 antitumor efficacy by promoting CD8+ progenitor exhausted T-cell expansion in tumor models. J Clin Invest. (2023) 133(7):e165673. doi: 10.1172/JCI165673 36853831 PMC10065084

[20] Scharping NE Rivadeneira DB Menk AV Vignali PDA Ford BR Rittenhouse NL. Mitochondrial stress induced by continuous stimulation under hypoxia rapidly drives T cell exhaustion. Nat Immunol (2021) 22(2):205–15. doi: 10.1038/s41590-020-00834-9 PMC7971090 33398183

[21] Deak LC Nicolini V Hashimoto M Karagianni M Schwalie PC Lauener L. PD-1-cis IL-2R agonism yields better effectors from stem-like CD8(+) T cells. Nature (2022) 610(7930):161–72. doi: 10.1038/s41586-022-05192-0 PMC9534752 36171284

[22] Scott AC Dundar F Zumbo P Chandran SS Klebanoff CA Shakiba M. TOX is a critical regulator of tumour-specific T cell differentiation. Nature (2019) 571(7764):270–4. doi: 10.1038/s41586-019-1324-y PMC7698992 31207604

[23] Liu BL Hu XD Feng KC Gao RR Xue ZQ Zhang SJ. Temporal single-cell tracing reveals clonal revival and expansion of precursor exhausted T cells during anti-PD-1 therapy in lung cancer. Nat Cancer. (2022) 3(1):108–21. doi: 10.1038/s43018-021-00292-8 35121991

[24] Wu TD Madireddi S de Almeida PE Banchereau R Chen YJJ Chitre AS. Peripheral T cell expansion predicts tumour infiltration and clinical response. Nature (2020) 579(7798):274–8. doi: 10.1038/s41586-020-2056-8 32103181

[25] Li Z Tuong ZK Dean I Willis C Gaspal F Fiancette R. *In vivo* labeling reveals continuous trafficking of TCF-1(+) T cells between tumor and lymphoid tissue. J Exp Med (2022) 219(6):e20210749. doi: 10.1084/jem.20210749 35472220 PMC9048291

[26] Rahim MK Okholm TLH Jones KB McCarthy EE Liu CC Yee JL. Dynamic CD8+ tcell responses to cancer immunotherapy in human regional lymph nodes are disrupted in metastatic lymph nodes. Cell (2023) 186(6):1127–1143.e18. doi: 10.1016/j.cell.2023.02.021 36931243 PMC10348701

[27] Martinez GJ Pereira RM Aijo T Kim EY Marangoni F Pipkin ME. The transcription factor NFAT promotes exhaustion of activated CD8(+) T cells. Immunity (2015) 42(2):265–78. doi: 10.1016/j.immuni.2015.01.006 PMC4346317 25680272

[28] Li HJ van der Leun AM Yofe I Lubling Y Gelbard-Solodkin D van Akkooi ACJ. Dysfunctional CD8 T cells form a proliferative, dynamically regulated compartment within human melanoma. Cell (2019) 176(4):775–789.e18. doi: 10.1016/j.cell.2018.11.043 30595452 PMC7253294

[29] Man K Gabriel SS Liao Y Gloury R Preston S Henstridge DC. Transcription factor IRF4 promotes CD8(+) T cell exhaustion and limits the development of memory-like T cells during chronic infection. Immunity (2017) 47(6):1129–41. doi: 10.1016/j.immuni.2017.11.021 29246443

[30] Seo H Chen J Gonzalez-Avalos E Samaniego-Castruita D Das A Wang YQH. TOX and TOX2 transcription factors cooperate with NR4A transcription factors to impose CD8(+) T cell exhaustion. Proc Natl Acad Sci U S A. (2019) 116(25):12410–12415. doi: 10.1073/pnas.1905675116 31152140 PMC6589758

[31] Chen ZY Ji ZC Ngiow SF Manne S Cai ZY Huang AC. TCF-1-Centered transcriptional network drives an effector versus exhausted CD8 T cell-fate decision. Immunity (2019) 51(5):840–855.e5. doi: 10.1016/j.immuni.2019.09.013 31606264 PMC6943829

[32] Ford BR Vignali PDA Rittenhouse NL Scharping NE Peralta R Lontos K. Tumor microenvironmental signals reshape chromatin landscapes to limit the functional potential of exhausted T cells. Sci Immunol (2022) 7(74):eabj9123. doi: 10.1126/sciimmunol.abj9123 35930654 PMC9851604

[33] Bejjani F Evanno E Zibara K Piechaczyk M Jariel-Encontre I. The AP-1 transcriptional complex: local switch or remote command? Biochim Biophys Acta-Rev Cancer (2019) 1872(1):11–23. doi: 10.1016/j.bbcan.2019.04.003 31034924

[34] Yang R Cheng SJ Luo N Gao RR Yu KZ Kang BX. Distinct epigenetic features of tumor-reactive CD8+T cells in colorectal cancer patients revealed by genome-wide DNA methylation analysis. Genome Biol (2019) 21(1):2. doi: 10.1186/s13059-019-1921-y 31892342 PMC6937914

[35] Hung MH Lee JS Ma C Diggs LP Heinrich S Chang CW. Tumor methionine metabolism drives T-cell exhaustion in hepatocellular carcinoma. Nat Commun (2021) 12(1):1455. doi: 10.1038/s41467-021-21804-1 33674593 PMC7935900

[36] Xu S Chaudhary O Rodriguez-Morales P Sun X Chen D Zappasodi R. Uptake of oxidized lipids by the scavenger receptor CD36 promotes lipid peroxidation and dysfunction in CD8+ tcells in tumors. Immunity (2021) 54(7):1561–1577.e7. doi: 10.1016/j.immuni.2021.05.003 34102100 PMC9273026

[37] Ma XZ Bi EG Lu Y Su P Huang CJ Liu LT. Cholesterol induces CD8(+) T cell exhaustion in the tumor microenvironment. Cell Metab (2019) 30(1):143–156.e5. doi: 10.1016/j.cmet.2019.04.002 31031094 PMC7061417

[38] Vardhana SA Hwee MA Berisa M Wells DK Yost KE King B. Impaired mitochondrial oxidative phosphorylation limits the self-renewal of T cells exposed to persistent antigen. Nat Immunol (2020) 21(9):1022–33. doi: 10.1038/s41590-020-0725-2 PMC7442749 32661364

[39] Yu YR Imrichova H Wang HP Chao T Xiao ZT Gao M. Disturbed mitochondrial dynamics in CD8(+)TILs reinforce T cell exhaustion. Nat Immunol (2020) 21(12):1540–51. doi: 10.1038/s41590-020-0793-3 33020660

[40] Lu D Liu L Sun YZ Song J Yin Q Zhang GZ. The phosphatase PAC1 acts as a T cell suppressor and attenuates host antitumor immunity. Nat Immunol (2020) 21(3):287–97. doi: 10.1038/s41590-019-0577-9 31932812

[41] Yau HL Ettayebi I De Carvalho DD. The cancer epigenome: exploiting its vulnerabilities for immunotherapy. Trends Cell Biol (2019) 29(1):31–43. doi: 10.1016/j.tcb.2018.07.006 30153961

[42] Ghoneim HE Fan YP Moustaki A Abdelsamed HA Dash P Dogra P. *De novo* epigenetic programs inhibit PD-1 blockade-mediated T cell rejuvenation. Cell (2017) 170(1):142–157.e19. doi: 10.1016/j.cell.2017.06.007 28648661 PMC5568784

[43] Belk JA Yao WN Ly N Freitas KA Chen YT Shi QM. Genome-wide CRISPR screens of T cell exhaustion identify chromatin remodeling factors that limit T cell persistence. Cancer Cell (2022) 40(7):768–786.e7. doi: 10.1016/j.ccell.2022.06.001 35750052 PMC9949532

[44] Shan Q Hu SE Chen X Danahy DB Badovinac VP Zang CZ. Ectopic Tcf1 expression instills a stem-like program in exhausted CD8(+) T cells to enhance viral and tumor immunity. Cell Mol Immunol (2021) 18(5):1262–77. doi: 10.1038/s41423-020-0436-5 PMC8093427 32341523

[45] Kamphorst AO Wieland A Nasti T Yang S Zhang R Barber DL. Rescue of exhausted CD8 T cells by PD-1-targeted therapies is CD28-dependent. Science (2017) 355(6332):1423–7. doi: 10.1126/science.aaf0683 PMC5595217 28280249

[46] Menk AV Scharping NE Rivadeneira DB Calderon MJ Watson MJ Dunstane D. 4-1BB costimulation induces T cell mitochondrial function and biogenesis enabling cancer immunotherapeutic responses. J Exp Med (2018) 215(4):1091–100. doi: 10.1084/jem.20171068 PMC5881463 29511066

[47] Duraiswamy J Turrini R Minasyan A Barras D Crespo I Grimm AJ. Myeloid antigen-presenting cell niches sustain antitumor T cells and license PD-1 blockade *via* CD28 costimulation. Cancer Cell (2021) 39(12):1623–1642.e20. doi: 10.1016/j.ccell.2021.10.008 34739845 PMC8861565

[48] Yost KE Satpathy AT Wells DK Qi YY Wang CL Kageyama R. Clonal replacement of tumor-specific T cells following PD-1 blockade. Nat Med (2019) 25(8):1251–9. doi: 10.1038/s41591-019-0522-3 PMC6689255 31359002

[49] Huang QZ Wu X Wang ZM Chen XY Wang LS Lu YJ. The primordial differentiation of tumor-specific memory CD8+T cells as bona fide responders to PD-1/PD-L1 blockade in draining lymph nodes. Cell (2022) 185(22):4049–4066.e25. doi: 10.1016/j.cell.2022.09.020 36208623

[50] Woroniecka KI Rhodin KE Dechant C Cui XY Chongsathidkiet P Wilkinson D. 4-1BB agonism averts TIL exhaustion and licenses PD-1 blockade in glioblastoma and other intracranial cancers. Clin Cancer Res (2020) 26(6):1349–58. doi: 10.1158/1078-0432.CCR-19-1068 PMC7073290 31871298

[51] Leem G Park J Jeon M Kim ES Kim SW Lee YJ. 4-1BB co-stimulation further enhances anti-PD-1-mediated reinvigoration of exhausted CD39(+) CD8 T cells from primary and metastatic sites of epithelial ovarian cancers. J Immunother Cancer. (2020) 8(2):e001650. doi: 10.1136/jitc-2020-001650 33335029 PMC7745695

[52] Salter AI Ivey RG Kennedy JJ Voillet V Rajan A Alderman EJ. Phosphoproteomic analysis of chimeric antigen receptor signaling reveals kinetic and quantitative differences that affect cell function. Sci Signal (2018) 11(544):eaat6753. doi: 10.1126/scisignal.aat6753 30131370 PMC6186424

[53] Edner NM Carlesso G Rush JS Walker LSK. Targeting co-stimulatory molecules in autoimmune disease. Nat Rev Drug Discovery (2020) 19(12):860–83. doi: 10.1038/s41573-020-0081-9 32939077

[54] Skokos D Waite JC Haber L Crawford A Hermann A Ullman E. A class of costimulatory CD28-bispecific antibodies that enhance the antitumor activity of CD3-bispecific antibodies. Sci Transl Med (2020) 12(525):eaaw7888. doi: 10.1126/scitranslmed.aaw7888 31915305

[55] Tolcher AW Sznol M Hu-Lieskovan S Papadopoulos KP Patnaik A Rasco D. Phase ib study of utomilumab (PF-05082566), a 4-1BB/CD137 agonist, in combination with pembrolizumab (MK-3475) in patients with advanced solid tumors. Clin Cancer Res (2017) 23(18):5349–57. doi: 10.1158/1078-0432.CCR-17-1243 28634283

[56] Weulersse M Asrir A Pichler AC Lemaitre L Braun M Carrie N. Eomes-dependent loss of the Co-activating receptor CD226 restrains CD8(+) T cell anti-tumor functions and limits the efficacy of cancer immunotherapy. Immunity (2020) 53(4):824–39.e10. doi: 10.1016/j.immuni.2020.09.006 33053331

[57] Liu C Somasundaram A Manne S Gocher AM Szymczak-Workman AL Vignali KM. Neuropilin-1 is a T cell memory checkpoint limiting long-term antitumor immunity. Nat Immunol (2020) 21(9):1010–21. doi: 10.1038/s41590-020-0733-2 PMC7442600 32661362

[58] Hanna BS Llao-Cid L Iskar M Roessner PM Klett LC Wong JKL. Interleukin-10 receptor signaling promotes the maintenance of a PD-1(i)(nt) TCF-1(+) CD8(+) T cell population that sustains anti-tumor immunity. Immunity (2021) 54(12):2825–2841.e10. doi: 10.1016/j.immuni.2021.11.004 34879221

[59] Kim CG Jang M Kim Y Leem G Kim KH Lee H. VEGF-a drives TOX-dependent T cell exhaustion in anti-PD-1-resistant microsatellite stable colorectal cancers. Sci Immunol (2019) 4(41):eaay0555. doi: 10.1126/sciimmunol.aay0555 31704735

[60] Zheng WT Wei J Zebley CC Jones LL Dhungana Y Wang YD. Regnase-1 suppresses TCF-1(+) precursor exhausted T-cell formation to limit CAR-t-cell responses against ALL. Blood (2021) 138(2):122–35. doi: 10.1182/blood.2020009309 PMC8288655 33690816

[61] Wu BG Zhang XW Chiang HC Pan HH Yuan B Mitra P. RNA Polymerase II pausing factor NELF in CD8(+) T cells promotes antitumor immunity. Nat Commun (2022) 13(1):2155. doi: 10.1038/s41467-022-29869-2 35444206 PMC9021285

[62] Chen J Lopez-Moyado IF Seo H Lio CWJ Hempleman LJ Sekiya T. NR4A transcription factors limit CAR T cell function in solid tumours. Nature (2019) 567(7749):530–4. doi: 10.1038/s41586-019-0985-x PMC6546093 30814732

[63] Tsao HW Kaminski J Kurachi M Barnitz RA DiIorio MA LaFleur MW. Batf-mediated epigenetic control of effector CD8(+) T cell differentiation. Sci Immunol (2022) 7(68):eabi4919. doi: 10.1126/sciimmunol.abi4919 35179948

[64] Seo H Gonzalez-Avalos E Zhang WD Ramchandani P Yang C Lio CWJ. BATF and IRF4 cooperate to counter exhaustion in tumor-infiltrating CAR T cells. Nat Immunol (2021) 22(8):983–95. doi: 10.1038/s41590-021-00964-8 PMC8319109 34282330

[65] Zhang XY Zhang CZ Qiao MM Cheng C Tang N Lu S. Depletion of BATF in CAR-T cells enhances antitumor activity by inducing resistance against exhaustion and formation of central memory cells. Cancer Cell (2022) 40(11):1407–1422.e7. doi: 10.1016/j.ccell.2022.09.013 36240777

[66] Liu Y Debo B Li MF Shi ZN Sheng WQ Shi Y. LSD1 inhibition sustains T cell invigoration with a durable response to PD-1 blockade. Nat Commun (2021) 12(1):6831. doi: 10.1038/s41467-021-27179-7 34819502 PMC8613218

[67] Qin Y Vasilatos SN Chen L Wu H Cao ZS Fu YM. Inhibition of histone lysine-specific demethylase 1 elicits breast tumor immunity and enhances antitumor efficacy of immune checkpoint blockade. Oncogene (2019) 38(3):390–405. doi: 10.1038/s41388-018-0451-5 30111819 PMC6336685

[68] Tu WJ McCuaig RD Tan AHY Hardy K Seddiki N Ali S. Targeting nuclear LSD1 to reprogram cancer cells and reinvigorate exhausted T cells *via* a novel LSD1-EOMES switch. Front Immunol (2020) 11:1228. doi: 10.3389/fimmu.2020.01228 32612611 PMC7309504

[69] Guo A Huang HL Zhu ZX Chen MJ Shi H Yuan SJ. cBAF complex components and MYC cooperate early in CD8(+) T cell fate. Nature (2022) 607(7917):135–41. doi: 10.1038/s41586-022-04849-0 PMC9623036 35732731

[70] Li J Wang WC Zhang YJ Cieslik M Guo JP Tan MY. Epigenetic driver mutations in ARID1A shape cancer immune phenotype and immunotherapy. J Clin Invest. (2020) 130(5):2712–26. doi: 10.1172/JCI134402 PMC7190935 32027624

[71] Gemta LF Siska PJ Nelson ME Gao X Liu XJ Locasale JW. Impaired enolase 1 glycolytic activity restrains effector functions of tumor-infiltrating CD8(+) T cells. Sci Immunol (2019) 4(31):eaap9520. doi: 10.1126/sciimmunol.aap9520 30683669 PMC6824424

[72] Guo YG Xie YQ Gao M Zhao Y Franco F Wenes M. Metabolic reprogramming of terminally exhausted CD8(+) T cells by IL-10 enhances anti-tumor immunity. Nat Immunol (2021) 22(6):746–56. doi: 10.1038/s41590-021-00940-2 PMC7610876 34031618

[73] Wenes M Jaccard A Wyss T Maldonado-Perez N Teoh ST Lepez A. The mitochondrial pyruvate carrier regulates memory T cell differentiation and antitumor function. Cell Metab (2022) 34(5):731–746.e9. doi: 10.1016/j.cmet.2022.03.013 35452600 PMC9116152

[74] Scharping NE Menk AV Whetstone RD Zeng X Delgoffe GM. Efficacy of PD-1 blockade is potentiated by metformin-induced reduction of tumor hypoxia. Cancer Immunol Res (2017) 5(1):9–16. doi: 10.1158/2326-6066.CIR-16-0103 27941003 PMC5340074

[75] Najjar YG Menk AV Sander C Rao U Karunamurthy A Bhatia R. Tumor cell oxidative metabolism as a barrier to PD-1 blockade immunotherapy in melanoma. JCI Insight (2019) 4(5):e124989. doi: 10.1172/jci.insight.124989 30721155 PMC6483505

[76] Lukhele S Abd Rabbo D Guo MD Shen J Elsaesser HJ Quevedo R. The transcription factor IRF2 drives interferon-mediated CD8+T cell exhaustion to restrict anti-tumor immunity. Immunity (2022) 55(12):2369–2385.e10. doi: 10.1016/j.immuni.2022.10.020 36370712 PMC9809269

[77] Benci JL Johnson LR Choa R Xu YM Qiu JY Zhou ZL. Opposing functions of interferon coordinate adaptive and innate immune responses to cancer immune checkpoint blockade. Cell (2019) 178(4):933–48. doi: 10.1016/j.cell.2019.07.019 PMC6830508 31398344

[78] Hashimoto M Araki K Cardenas MA Li P Jadhav RR Kissick HT. PD-1 combination therapy with IL-2 modifies CD8(+) T cell exhaustion program. Nature (2022) 610(7930):173–81. doi: 10.1038/s41586-022-05257-0 PMC9793890 36171288

[79] Liu YY Zhou NN Zhou L Wang J Zhou YB Zhang TZ. IL-2 regulates tumor-reactive CD8(+) T cell exhaustion by activating the aryl hydrocarbon receptor. Nat Immunol (2021) 22(3):358–69. doi: 10.1038/s41590-020-00850-9 33432230

[80] Arenas-Ramirez N Zou C Popp S Zingg D Brannetti B Wirth E. Improved cancer immunotherapy by a CD25-mimobody conferring selectivity to human interleukin-2. Sci Transl Med (2016) 8(367):367ra166. doi: 10.1126/scitranslmed.aag3187 27903862

[81] Bae J Liu LC Moore C Hsu E Zhang AL Ren ZH. IL-2 delivery by engineered mesenchymal stem cells re-invigorates CD8(+) T cells to overcome immunotherapy resistance in cancer. Nat Cell Biol (2022) 24(12):1754–65. doi: 10.1038/s41556-022-01024-5 36474070

[82] Chen WX Teo JMN Yau SW Wong MYM Lok CN Che CM. Chronic type I interferon signaling promotes lipid-peroxidation-driven terminal CD8(+) T cell exhaustion and curtails anti-PD-1 efficacy. Cell Rep (2022) 41(7):111647. doi: 10.1016/j.celrep.2022.111647 36384131

[83] Naing A Infante JR Papadopoulos KP Chan IH Shen C Ratti NP. PEGylated IL-10 (Pegilodecakin) induces systemic immune activation, CD8(+) T cell invigoration and polyclonal T cell expansion in cancer patients. Cancer Cell (2018) 34(5):775–91. doi: 10.1016/j.ccell.2018.10.007 PMC8098754 30423297

[84] Naing A Papadopoulos KP Autio KA Ott PA Patel MR Wong DJ. Safety, antitumor activity, and immune activation of pegylated recombinant human interleukin-10 (AM0010) in patients with advanced solid tumors. J Clin Oncol (2016) 34(29):3562–9. doi: 10.1200/JCO.2016.68.1106 PMC5657013 27528724

[85] Naing A Wong DJ Infante JR Korn WM Aljumaily R Papadopoulos KP. Pegilodecakin combined with pembrolizumab or nivolumab for patients with advanced solid tumours (IVY): a multicentre, multicohort, open-label, phase 1b trial. Lancet Oncol (2019) 20(11):1544–55. doi: 10.1016/S1470-2045(19)30514-5 PMC8436252 31563517

